# miR-145 restoration overcomes oxaliplatin resistance via ABCC1 in colorectal cancer

**DOI:** 10.1186/s43046-026-00349-8

**Published:** 2026-05-25

**Authors:** Mahsa Sadeghzadeh, Milad Asadi, Behzad Baradaran, Venus Zafari, Soghra Bornehdeli, Haniye Mohammad Reza Khani, Behzad Mansoori, Habib Zarredar, Dariush Shanehbandi

**Affiliations:** 1 Immunology Research Center, Tabriz University of Medical Sciences, Tabriz, Iran; 2 Department of Basic Oncology, Institute of Health Sciences, Ege University, Izmir, Turkey; 3 Tuberculosis and Lung Diseases Research Center, Tabriz University of Medical Sciences, Tabriz, Iran

**Keywords:** Colorectal cancer, SW-480 cells, Oxaliplatin, Drug resistance, microRNA-145, Apoptosis, MDR1, ABCC1

## Abstract

**Background:**

Drug resistance limits effectiveness of chemotherapy in colorectal cancer (CRC). Consequently, finding appropriate strategies for re-sensitizing chemo-resistant cells is crucial. Our present study aimed at taking advantage of chemotherapy and gene therapy by restoring microRNA-145 expression in oxaliplatin resistant CRC cells.

**Methods:**

Bioinformatic analysis of clinical CRC datasets demonstrated significantly reduced miR-145 expression in tumor tissues compared with normal samples. Oxaliplatin-resistant SW-480 cells were generated and transfected with a pCMV-miR-145 expression vector. The MTT assay was used to assess cell viability following miR-145 restoration, oxaliplatin treatment, or their combination. Apoptosis was measured via flow cytometry. Gene expression levels of ABCC1, MDR1, K-RAS, MMP-13, Bcl-2, CASP3, CASP8, and CASP9 were analyzed using qRT-PCR, and ABCC1 protein expression was evaluated by western blotting. Cell migration was assessed using a wound-healing assay.

**Results:**

Co-treatment with miR-145 and Oxaliplatin significantly reduced cell viability, proliferation, and migration and increased apoptosis compared to the either treatments. Restoring miR-145 expression downregulated reduced the drug-resistance genes ABCC1 and MDR1, and reduced decreased expression of oncogenes including K-RAS and Bcl-2, while increasing expression of apoptosis-related genes (CASP3, CASP8, and CASP9).

**Conclusion:**

miR-145 restoration via decreasing the drug resistance biomarkers ABBCC1 and MDR1, along with other oncogenes like K-Ras and Bcl2, and increasing apoptosis conductors could sensitize oxaliplatin-resistant cells to chemotherapy. This proposes a novel and clinically translatable strategy to control drug resistance in CRC, finding new ways to increasing chemotherapeutic efficacy.

**Supplementary Information:**

The online version contains supplementary material available at 10.1186/s43046-026-00349-8.

## Introduction

One of the most prevalent cancers of the digestive system, colorectal cancer (CRC) is the third most common cause of cancer-related death in the world [1]. Drug resistance is a main challenge in colorectal cancer therapy, with mechanisms including changing in non-coding RNAs leading to significantly to therapeutic failure. Recent research have highlighted the role of small RNAs, including microRNAs (miRNAs) and tRNA-derived small RNAs (tsRNAs), in controlling drug resistance via modulating key signaling pathways and drug transporters [2, 3]. Despite significant advances in CRC diagnosis and treatment, survival rates in late-stage disease are poor due to drug resistance and chemotherapy failures [4, 5]. Oxaliplatin is a platinum-based chemotherapeutic agent that usually combined with other chemotherapy drugs (such as Irinotecan, 5-FU, and leucovorin), as standard treatment for stage II and stage III CRC [6, 7]. However, the problem is acquired drug resistance limits the effectiveness of this agent as a cancer treatment. The lack of knowledge of the mechanisms responsible for drug resistance hampers efforts to develop effective therapeutics [8].

Recently, combination of chemotherapy and gene-based therapy have been proposed as a cancer treatment [9]. A recent strategy in this area is microRNA replacement therapy, which introduces tumor-suppressor miRNAs into malignant cells [10].microRNAs (miRNAs) are categorized as oncogenic and tumor suppressor microRNAs [11]. They are important regulators of several cellular processes, such as cell proliferation, apoptosis, and differentiation [12]. Dysregulation of miRNA expression is found in a wide range of human cancers. A decrease in the expression level of tumor suppressor miRNAs can result in uncontrolled growth via upregulation of oncogenes and anti-apoptotic genes [13, 14]. The miR-145 is significantly down-regulated in several types of cancer, including colorectal cancer, kidney, esophageal, bladder, and breast cancer [11, 15]. MiR-145 regulates various essential cellular events, including proliferation, differentiation, apoptosis, invasion and metastasis [11, 16]. Specifically, miR-145 can increase the sensitivity of cancer cells to therapeutic agents by targeting important resistance-associated genes, such as Bcl-2, and ABCC1 [8, 15, 17, 18]. Although miR-145 functions as a tumor suppressor, its role in reversing oxaliplatin resistance in colorectal cancer especially through regulation of drug-efflux transporters like ABCC1 remains unclear. We hypothesized that restoring miR-145 in oxaliplatin-resistant CRC cells would sensitize them to treatment by suppressing ABCC1 and other oncogenic pathways.

## Methods

### Cell culture

The human colorectal cancer cell line (SW-480) was purchased from the Pasteur Institute, Iran and cells were cultured in Roswell Park Memorial Institute (RPMI)-1640 medium containing 10% fetal bovine serum (Gibco Laboratories, Grand Island, NY), 100 IU/ml penicillin and 100 µg/ml streptomycin. Cultures were incubated at a 37 °C incubator (Memmert, Schwabach, Germany) with 5% CO_2_ and a humidified atmosphere. In all experimental procedures, cells were used in a logarithmic growth phase. The oxaliplatin-resistant SW-480 cells were generated by exposing the parental cells to increasing concentrations of oxaliplatin. The initial dose for this purpose was 0.5 µM (cells were treated with this concentration for 60 days then concentration gradually increase 0.1 µM every week up to 2µM) that reached 2µM after 5 months.

### Bioinformatics analysis

The Cancer Genome Atlas (TCGA) dataset was used to evaluate MiR-145 expression levels in 251 colon adenocarcinomas and 7 normal tissues. The data were analyzed by R using limma, and GEOquery package. In the context of clinical information, miR-145 expression levels were determined in colorectal primary tumors, individual cancer stages, and nodal metastases. A P-value of < 0.05 compared to normal samples was considered statistically significant. Potential targets of miR-145 were predicted using the online prediction tools, Target scan, miRWalk and miRmap.

### Plasmid vector preparation

**Fig. 1:**
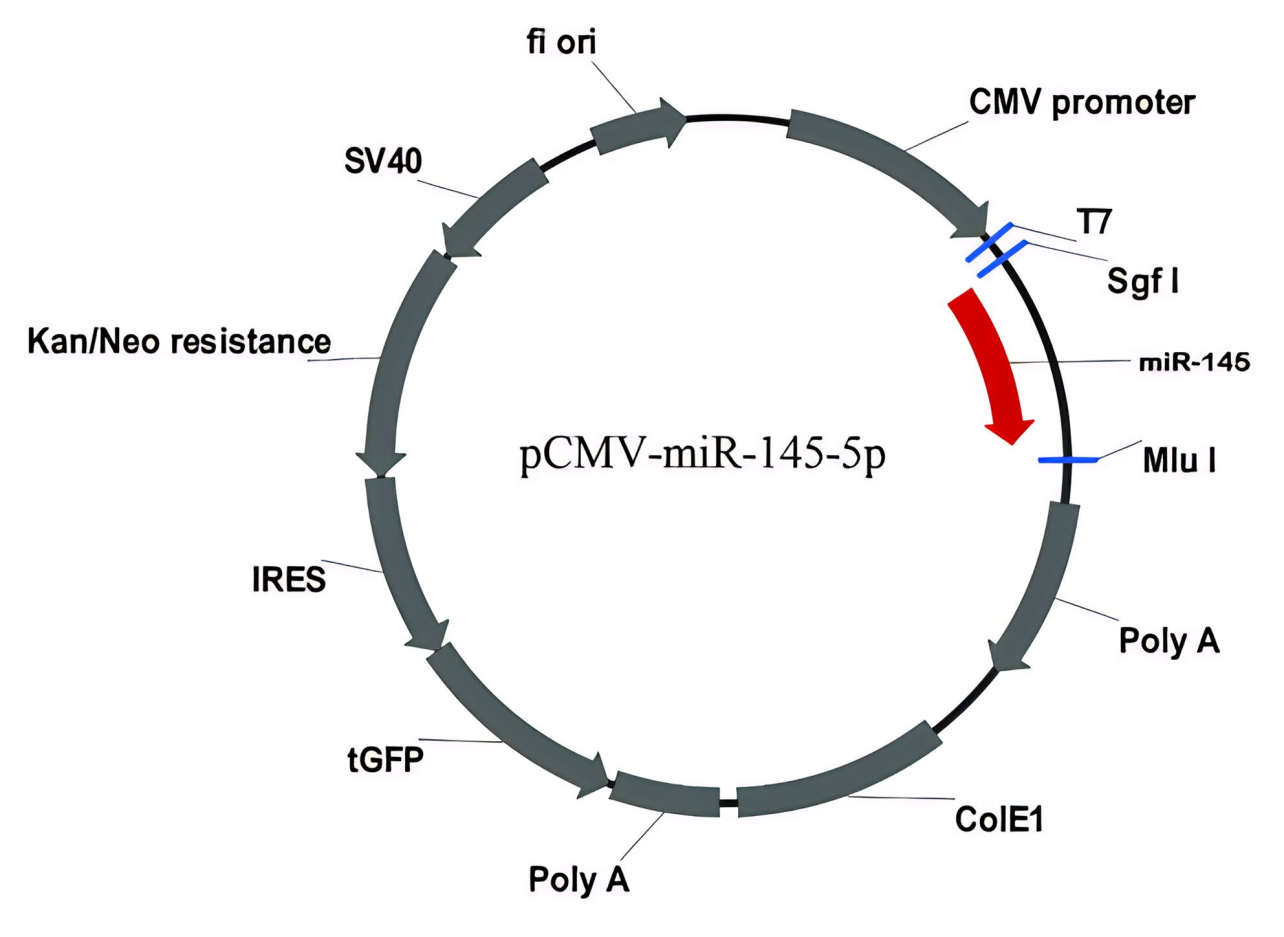
Schematic representation of the pCMV-miR-145 expression vector. The map illustrates the main functional elements of the pCMV-miR-145 construct used to overexpress miR-145 in colorectal cancer cells

A plasmid vector encoding miR-145 (Fig. 1) was obtained from OriGene Company. Empty pCMV vector was used as the negative control. Both pCMV-miR-145 (vec+) and empty pCMV (vec−) vectors contained G-418 (Geneticin) resistance, as well as GFP coding sequence for selection and screening purposes. *Escherichia coli* (TOP10 strain).

was transformed as a host for plasmid proliferation by the method described before [19]. Briefly, *E. coli* bacteria were cultured in LB broth medium. When the optical density (OD600) reached 0.9, bacteria were harvested by centrifugation at 1000 g. Afterwards, competent cells were prepared by treating the bacteria with an ice-cold solution of 80 mM CaCl_2_ and 20 mM MgCl_2_. The transformation was done by 10 min of vibrating at 1000 rpm at 37 °C using an “Eppendorf thermomixer C”. Transformed cells were plated on LB agar plates containing 30 µg/ml kanamycin were used for screening of positive colonies. Positive colonies expressing GFP were selected, and plasmid Maxi Kit (QIAGEN, 12162) was utilized for plasmid DNA extraction from the confirmed colonies. 

### Transfection of SW-480 cells

**Fig. 2:**
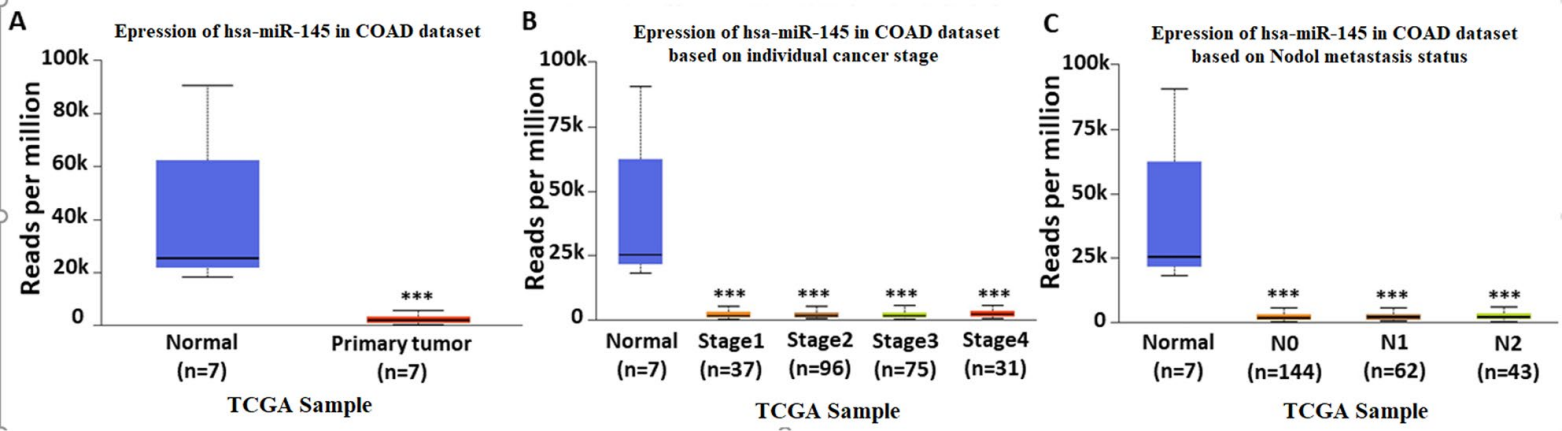
miR-145 expression reduced in colon adenocarcinoma. Expression data from the TCGA-COAD project show that miR-145 levels are significantly decreased in primary tumor tissues compared with normal colon tissues (**A**). miR-145 expression is consistently reduced across tumor stages I–IV (**B**) and across nodal status groups N0–N3 compared with normal tissues (**C**). ***P < 0.001 versus normal tissues

Transfection was performed by electroporatino (Gene Pulser Xcell^(TM)^, Bio-rad, USA) and a quantity of 5 × 10^5^ oxaliplatin resistant SW-480cells were resuspended in electroporation buffer. Electcroporation buffer contained HEPES 21 mM, NaCl 137 mM, KCL 5 mM, Na_2_HPO_4_, 7H_2_O 0.7 mM and Dextrose 6 mM. A single 200 V pulse, with a duration of 25 ms, was utilized for transfection of 10 µg of the pCMV-miR-145 vector and the same protocol used for blank pCMV vector (negative control). After 24 h, cells were monitored by a live cell imaging system (Cytation 5, Biotek, Winooski, VT) for GFP expression and successful transfection (Fig. 2A).

Moreover, restoration of miR-145 expression was confirmed by quantitative real-time PCR (qRT-PCR). For this purpose, total RNA was extracted using the RiboEx reagent (GeneAll biotechnology, Seoul, Korea) according to the manufacturer’s instructions. The quality and concentration of the extracted RNA was assessed by gel electrophoresis and NanoDrop OneCspectrophotometer (Thermo Scientific, USZ). The cDNA for miRNA quantification was synthesized from 10ng of the total RNA using the universal cDNA synthesis Kit (Exiqon, Vedbæk, Denmark). The qRT-PCR was performed by SYBR Green method and miR-145 specific primers (Exiqon, Denmark) using LightCycler 96 equipment (Roche Diagnostics, Mannheim, Germany). The two-step PCR comprised 45 cycles of 96 °C for 10 s and 60 °C for 1 min. The expression of miR-145 was assessed and compared with the control group (transfected with an empty vector) using the (2^−ΔΔCT^) formula. Normalization was performed using U6. 

### Cell viability assay

The MTT assay was employed for the assessment of cell cytotoxicity following the individual miR-145 restoration, oxaliplatin treatment and combination of both oxaliplatin treatment and miR-145 overexpression. MTT assay was performed by seeding 3 × 10^3^ oxaliplatin resistant cells in 96 well culture plates and transfecting the cells with miR-145. Since continuous exposure to oxaliplatin was required to maintain the chemoresistant phenotype, cells have to be treated with oxaliplatin regularly in order to simulate chemo-resistance. Nevertheless, to decrease the electroporation-induced cellular stress, oxaliplatin treatment was delayed for 48 h after transfection to give cells time to recover. Afterwards, cells were treated with the same concentration of this drug (2ng/ml). After 24 h of oxaliplatin treatment, the effect of miR-145 restoration on the viability of the oxaliplatin resistant cells was assessed by 3-(4, 5-dimethylthiazol-2-yl)-2, 5-diphenyl tetrazolium bromide (MTT) assay. MTT powder (Sigma, Taufkirchen, Germany) was used as a reagent at a concentration of 0.02 mg/ml (in RPMI-1640 medium containing 10% FBS). Then, cells were placed in a 37 °C incubator. After 4 h, the medium was replaced with 100 µl of dimethyl sulfoxide (DMSO) and 25 µl of Sorenson’s buffer (glycine 0.1 M, NaCl 0.1 M, pH: 10.5 with 0.1 NaOH pH 10.5). The plate was gently shaken and then incubated at 37 °C for 30 min to fully dissolve. The plate was gently shaken and then incubated at 37 °C for 30 min to fully dissolve the formazan crystals. 

### Apoptosis assay

The annexin V/propidium iodide (PI) flow cytometry assay was used to measure apoptosis induction following individual miR-145 overexpression, oxaliplatin treatment and the combined treatment. A total of 1 × 10⁵ cells per well were seeded into 6-well plates and divided into four groups: (i) miR-145 overexpression, (ii) oxaliplatin treatment, (iii) combined miR-145 + oxaliplatin treatment, and (iv) negative control (vec−). Oxaliplatin-resistant cells transfected with empty vector (vec−) were considered as a negative control. A total of 1 × 10^5^ oxaliplatin resistant cells were seeded into 6-well plates, and divided into four groups: (i) miR-145 overexpression, (ii) oxaliplatin treatment, (iii) combined miR-145 + oxaliplatin treatment, and (iv) negative control (vec−). The subject was then treated with a chemotherapy agent and 24 h later, the cells were trypsinized and centrifuged at 1,500 g for 5 min. Next, the cells were stained with Annexin V and PI in accordance with the manufacturer’s instructions (Roche). Briefly, the samples were incubated with 2 ml of annexin V, 1 ml of propidium iodide, and 100 ml of binding buffer. Afterwards, the cells were incubated for 15 min at room temperature under dark conditions. The samples were analyzed using a flow cytometry instrument (Macs Quant Analyser 10, Miltenyi Biotech, Germany). FlowJo software (Tree Star, San Carlos, CA) was used to assess the rate of apoptosis. 

### Wound healing assay

For evaluating cell migration in treated groups, we used a wound-healing (scratch) assay. Oxaliplatin exposure was temporarily suspended prior to transfection. Approximately 48 h after electroporation, the chemotherapy agent was added at a dose of 1ng/ml. Oxaliplatin-resistant SW-480 cells were seeded in 24 well plates at a density of 25 × 10 ^3^ cells, and once they achieved a confluency of 80%-90%, a yellow tip was used to generate wound gaps in cell monolayers. We monitored cell migration to fill the gaps after removing detached cells with fresh RPMI-1640 medium. Images of the wound area were captured at 0, 24, 48, and 72 h to evaluate migration. 

### Assessment of mRNA expression by quantitative real-time PCR (qRT-PCR)

A qRT-PCR assay was employed to evaluate the expression levels of ABCC1, MDR1, K-RAS, MMP-13, Bcl-2, CASP-3, CASP-8 and CASP-9 in the treatment groups miR-145 overexpression, Oxaliplatin treatment, and their combination). A number of 5 × 10^5^ oxaliplatin-resistant cells were electroporated and allowed to recovered for 48 h in absence of a chemotherapy agent in a 25cm^2^ culture flask. After recovery, cells received the designated treatments, and 24 h later, total RNA was extracted, using RiboEx (GeneAll, Seoul, Korea). Also, the quantity and quality of the extracted total RNA was evaluated by Nanodrop 2000 system (Thermo Fisher Scientific, Wilmington, DE, USA). Complementary DNA (cDNA) was synthesized using 1 µg of RNA template by Revert Aid cDNA Synthesis kit (Thermo, USA). PCR conditions were an enzyme activation step of 95 °C followed by 45 cycles of denaturing at 95 °C for 10 s, annealing at 60 °C for 30 s, and extension at 72 °C for 20 s. Real-time PCR was performed using SYBR Green PCR Pre-Mix (Ampliqon, Denmark) and GAPDH was used as the housekeeping gene. The expression levels were quantified using light cycler systems 96 (Roche, Germany) equipment. Relative mRNA expression levels were calculated using the 2⁻^ΔΔCt^ (Livak) method. The primer sets used for the quantification of target genes are provided in Table 1 with more details.

**Table 1:** Primer sequences

Gene		Primer sequence
GAPDH	Forward Reverse	5′-CAAGATCATCACCAATGCCT‐3′ 5′-CCCATCACGCCACAGTTTCC‐3′
BCL-2	Forward Reverse	5′-GAGCGTCAACCGGGAGATGTC‐3′ 5′-TGCCGGTTCAGGTACTCAGTC‐3′
ABCC1	Forward Reverse	5′-AGAACAAGACGCGGATCTTGG‐3′ 5′- GCATAGGTACGCAGGAACTCA‐3′
KRAS	Forward Reverse	5′-GCCTGCTGAAAATGACTGAATATA-3′ 5′-TTAGCTGTAATCGTCAAGGCACTC − 3′
MDR1	Forward Reverse	5′-CCCATCATTGCAATAGCAGG-3′ 5′-TGTTCAAACTTCTGCTCCTGA − 3′
Caspase3	Forward Reverse	5ʹ-ATGGTTTGAGCCTGAGCAGA-3ʹ 5ʹ-GGCAGCATCATCCACACATAC-3ʹ
Caspase8	Forward Reverse	5ʹ-ACCTTGTGTCTGAGCTGGTCT-3ʹ 5ʹ-GCCCACTGGTATTCCTCAGGC − 3ʹ
Caspase9	ForwardReverse	5ʹ-GCAGGCTCTGGATCTCGGC-3ʹ5ʹ-GCTGCTTGCCTGTTAGTTCGC-3ʹ
MMP13	Forward Reverse	5ʹ- GACAAGTAGTTCCAAAGGCTACAA-3ʹ 5ʹ-GGGTTGGGGTCTTCATCTC-3ʹ

### Western blot analysis

Western blotting was performed to determine whether changes in ABCC1 protein levels is related to miR-145 expression,. For this purpose, 1 × 10^5^ oxaliplatin resistant SW-480 cells were transfected and seeded into a 6-well culture plate. Oxaliplatin treatment was continued 48 h following electroporation. After that, cells were detached and lysed in RIPA buffer (Santa Cruz Biotech, USA) for protein extraction. A Bradford Protein assay kit (Thermo Fisher Scientific, Cat. No. 23200) was utilized to quantify the protein concentration, and a 12.5% SDS polyacrylamide gel was used to separate 25 µg of the protein lysate. The protein bands were transferred onto polyvinylidene difluoride membranes (Millipore, USA) using a semi-dry electroblotting system (Bio-Rad Laboratories, USA). Membranes were blocked with 3% bovine serum albumin (BSA), and subsequently membranes were incubated with primary mouse monoclonal antibodies against ABCC1 (1:1000, IU2H10, Novus Biologicals) and β-actin (1:1000, ab8227, Abcam) as the normalizing control. After washing, membranes were incubated with horseradish peroxidase-linked goat anti-mouse antibody (1:3000, Abcam) for one hour at room temperature. To visualize bands, a chemiluminescence detection kit (Roche Diagnostics GmbH, Germany) was used with a gel documentation system.

### Statistical analysis

The statistical analysis was performed using GraphPad Prism (version 6. 0, San Diego, CA). All data were stated as means ± SEM (standard error mean). All experiments were performed independently at least three times. Student’s t and analysis of variance (ANOVA) tests were exploited to determine the statistical significance of differences and p values < 0. 05 were considered significant.

## Results

### hsa-miR-145 prevented the proliferation of oxaliplatin resistant SW-480 cells

In colon adenocarcinomas samples, miR-145 expression was significantly decreased in primary tumors compared to normal tissues (Fig. 2A). Additionally, miR-145 expression levels were significantly lower in stages (I–IV) as well as N0-N2 nodal metastasis compared to normal tissues (Fig. 2B and C). Although miR-145 was considerably down regulated in all tumor nodal subgroups (N0–N2) compared with normal tissues (*** *P* < 0.001), we did not observe statistically significant differences among N0, N1, and N2. This shows that miR-145 suppression happens early and remains relatively stable across nodal stages progresses. 

### Transfection of the oxaliplatin resistant CRC cells

**Fig. 3:**
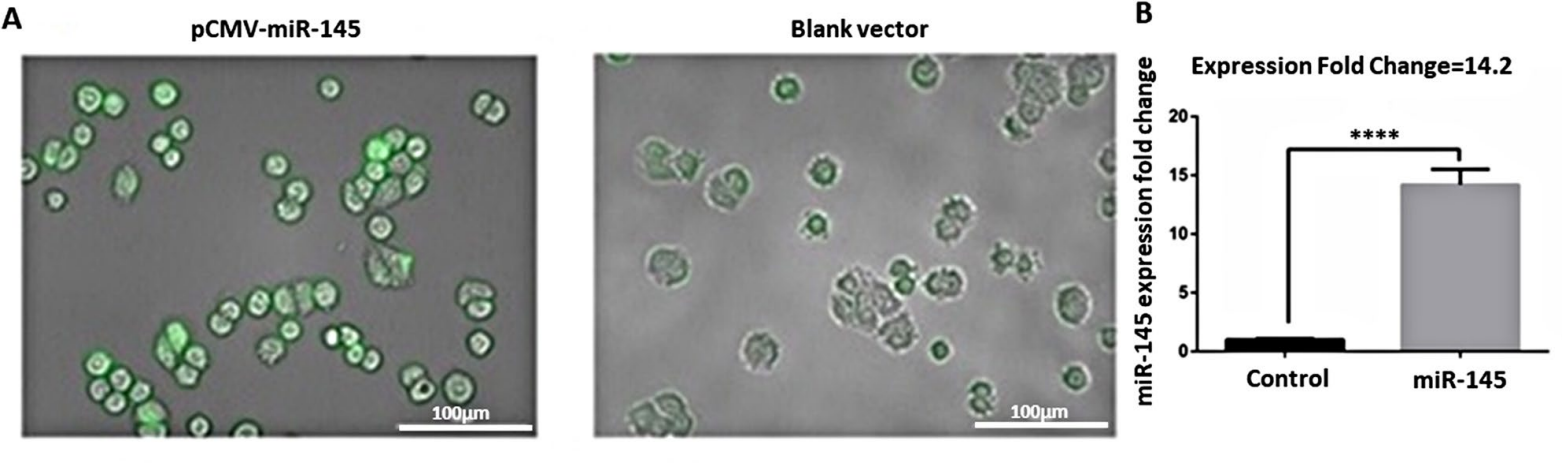
pCMV-miR-145 induces miR-145 expression in oxaliplatin-resistant colorectal cancer cells. GFP expression in oxaliplatin-resistant cells transfected with pCMV-miR-145 (left) or empty vector (right) confirms successful transfection (**A**). miR-145 expression was increased more than 14-fold in cells transfected with pCMV-miR-145 compared with cells transfected with the empty vector (*P* < 0.0001) (**B**)

The microRNA expression vector backbone contained a GFP reporter sequence. Thus, the successful transfection of the CRC cells was confirmed by the expression of GFP as shown (Fig. 3A). Additionally, miRNA analysis revealed a 14.2fold increase in miR-145 expression in cells transfected with the pCMV-miR-145 vector compared to the blank control (Fig. 3B).

### MiR-145 restoration leads to sensitization of the oxaliplatin resistant colorectal cancer cells

**Fig. 4:**
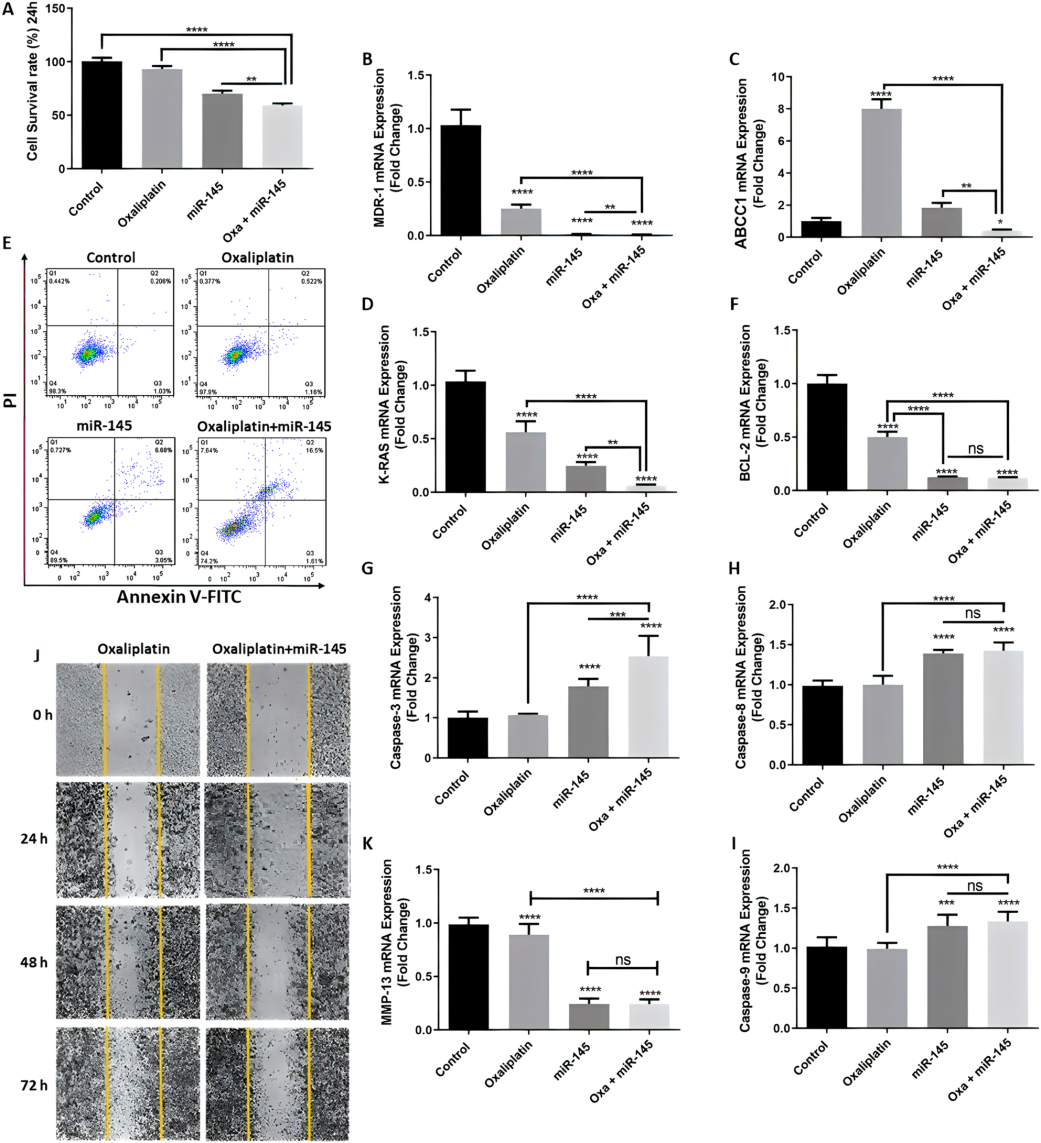
miR-145 monotherapy

The effects of the miR-145 overexpression on drug sensitivity was detected using the MTT assay. As shown in Fig. 4A, the proliferation rate of cells treated with the miR-145 vector and Oxaliplatin combination significantly decreased compared with the control group (*p* < 0.0001), cells treated with Oxaliplatin alone (*p* < 0.0001) and cells treated with miR-145 alone (*p* = 0.0035). Correspondingly, according to the results of qRT-PCR, the expression level of MDR-1, ABCC1 and K-RAS in the group treated with the combination of miR-145 and Oxaliplatin had significantly decreased in comparison with the cells treated with oxaliplatin alone (*p* < 0.0001) (Fig. 4B and D). To evaluate the miR-145’s impact on the apoptotic rate of oxaliplatin-resistant SW-480 cells, an annexin V/PI flow cytometry assay was employed. Flow cytometry results have indicated that the percentage of late apoptosis in the group treated with combined miR-145 and Oxaliplatin was markedly higher than oxaliplatin mono-therapy group (1.682% vs. 18.11%). Together, these studies showed that miR-145 overexpression significantly reduced the resistance of Oxaliplatin resistant SW-480 cells to oxaliplatin (Fig. 4E). These results were supported with the increased mRNA expression levels of CASP-3, -8, -9, and Bcl-2 genes. A significant down regulation in Bcl-2 mRNA expression was observed in the group treated with a combination of miR-145 and Oxaliplatin compared with the control group (*p* = 0.0001) and the group treated with only Oxaliplatin (*p* = 0.0001). However, there was no difference compared with the group treated with only miR-145 (*p* = 0.237) (Fig. 4F). There was a significant increase in mRNA expression of CASP-3, CASP-8, and CASP-9 in the combination group in comparison to the other groups. Specifically, CASP-3 mRNA expression was considerably up regulated in the group treated with a combination of miR-145 and oxaliplatin in comparison to the control group (*p* < 0.0001), the group treated with only miR-145 (*p* = 0.00081), and the group treated with only oxaliplatin (*p* = 0.00081).

Additionally, the expression level of CASP-8 and CASP-9 mRNA in the combination of miR-145 and oxaliplatin were significantly increased in comparison to the control group (*p* < 0.0001) and the group treated with only oxaliplatin (*p* < 0.0001). However, there was no significant change in comparison to the group treated with only miR-145 (*p* = 0.451 and *p* = 0.279, respectively) (Fig. 4G and I). To evaluate the inhibitory effect of miR-145 on the migration of oxaliplatin-resistant CRC cells, a wound-healing (scratch) assay was employed. Oxaliplatin- resistant SW-480 cells treated with combined oxaliplatin and miR-145 were compared with cells exposing empty vectors (negative control) and oxaliplatin. Compared to the controls, cells overexpressing miR-145 showed a significant decrease in migration in 24, 48, and 72 h (Fig. 4J). Consequently, qRT-PCR analysis showed significant reduction in MMP-13 mRNA expression levels in the combined miR-145-oxaliplatin treatment compared to the control group (*p* < 0.0001) and the group exposed to oxaliplatin alone (Fig. 4K). It was not significantly different between groups treated with miR-145 alone versus those treated with the combination (*p* = 0.91). 

### Bioinformatics analysis and protein expression of drug resistance gene

**Fig. 5:**
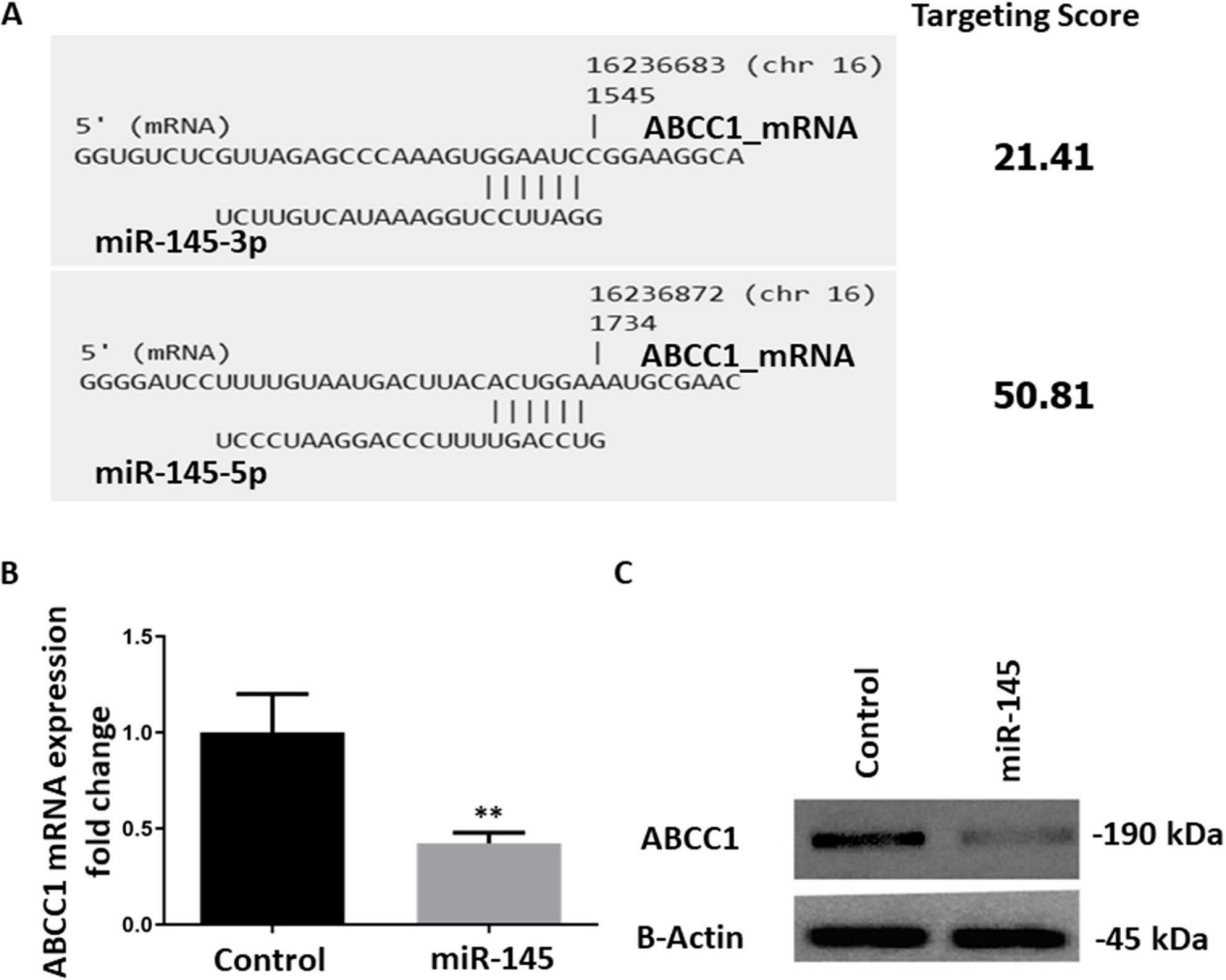
miR-145 targets ABCC1 mRNA**.** Target prediction analysis showed both miR-145-3p and − 5p recognize binding sites within ABCC1 mRNA, with prediction scores of 21.41 and 50.81, respectively (**A**). ABCC1 mRNA expression was significantly reduced in cells transfected with miR-145 compared with the blank control (**B**). ABCC1 protein levels were decreased in the group receiving combined oxaliplatin and miR-145 treatment compared with the blank control, with β-actin used as a loading control (**C**). ****P < 0.0001

**Fig. 6:**
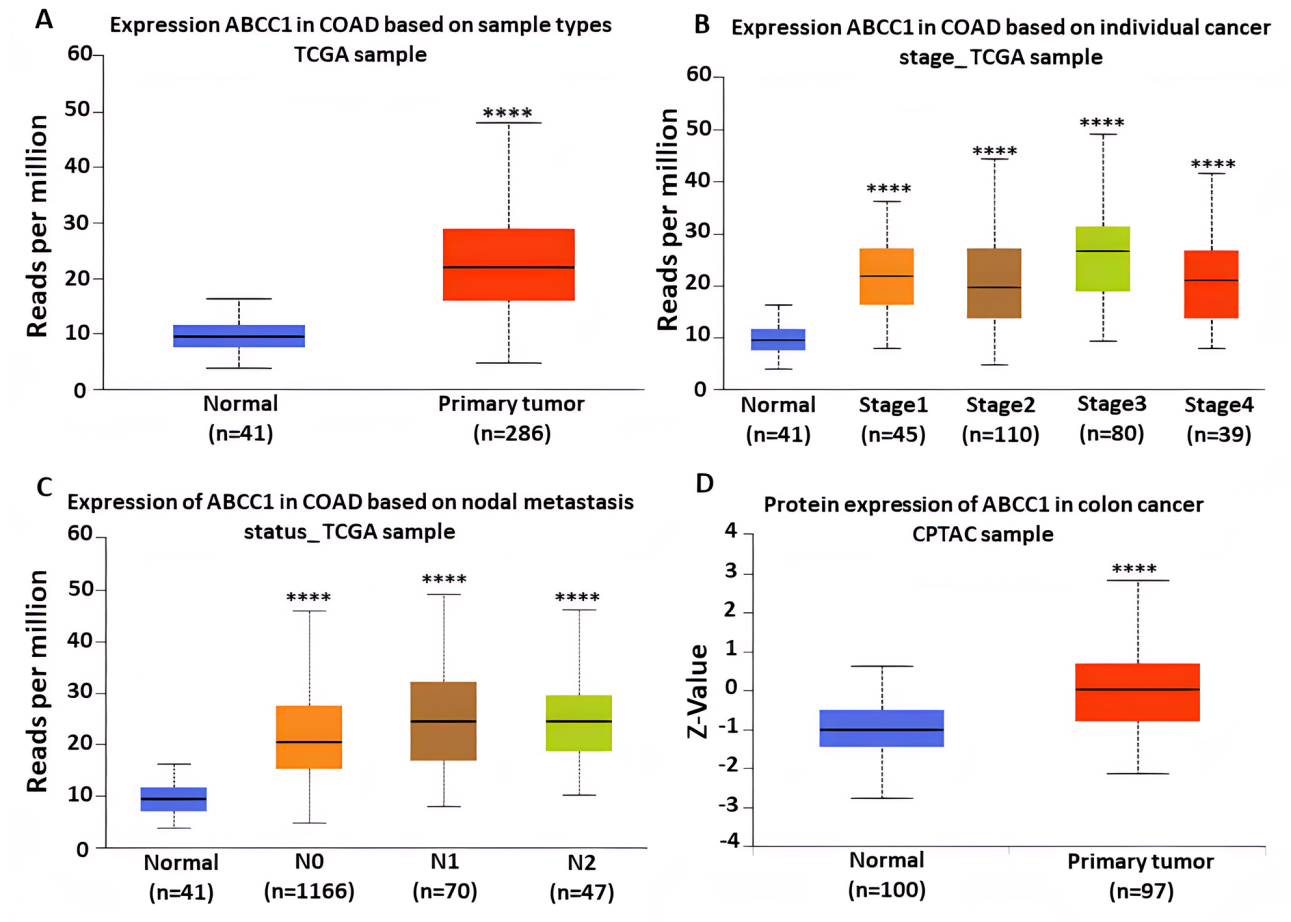
ABCC1 overexpressed in colon adenocarcinoma. Data from the TCGA-COAD project shows that ABCC1 expression is significantly increased in primary tumors tissues compared with adjacent normal tissues (**A**). ABCC1 expression level significantly increased across tumor stages I–IV (**B**), and N0-N2 tissues compared to adjusted normal tissues (**C**). ABCC1 Protein expression was increased in colon cancer tissues compared to adjusted normal tissues from TCGA and the Cancer Institute’s Clinical Proteomic Tumor Consortium (CPTAC) samples (**D**). ****P < 0.0001

A bioinformatics target prediction analysis indicated that miR-145-3p and − 5p may directly target ABCC1 mRNA, with prediction miRmap scores of 21.41 and 50.81, respectively (Fig. 5A). A significant reduction in ABCC-1 protein expression occurred after miR-145 transfection in comparison to the control group (Fig. 5B and C). Further bioinformatics analysis of ABCC1 levels in the clinical colorectal samples of the COAD project (TCGA dataset) revealed ABCC1 overexpression in primary tumors (Fig. 6A), across stage I–IV (Fig. 6B), and in nodal metastasis groups (N0-N2) colorectal tumor samples (Fig. 6C). Additionally, based on the data of the Cancer Institute’s Clinical Proteomic Tumor Consortium (CPTAC), the protein expression level of ABCC1 increased at a significant rate in colorectal cancer samples (Fig. 6D).

## Discussion

Globally, CRC is one of the most prevalent malignancies of the digestive system and remains a major cause of cancer-related mortality. Although there has been significant progress in the diagnosis and treatment of CRC, there still exists a low survival rate of cancer patients. In this study, we demonstrate that miR-145 sensitizes oxaliplatin-resistant SW-480 cells to oxaliplatin. There have been several studies that suggest miR-145 could plays a tumor-suppressive role and may reduce chemoresistance in various cancers [8, 20]. Oxaliplatin is a widely used chemotherapy agent for colorectal cancer and several other malignancies, either as monotherapy or in combination with other therapeutics such as Fluorouracil [21]. However, acquired drug resistance is one of the most common limiting factors in the treatment of CRC. To overcome this limitation, combinational strategies such as chemo/gene therapy (like miRNA-based approaches) have emerged to improve therapeutic effectiveness and remedy the existing disadvantages [22]. In our study, bioinformatics analysis revealed that miR-145 was considerably downregulated in colorectal cancer tissues compared with normal tissues. It has been reported that miR-145 has been reduced in precancerous colorectal lesions as well as in different stages of colorectal cancer tissue samples compared to normal colorectal mucosa [23]. Several studies have suggested that the miRNA expression profiles of 31 pairs CRC tissues and adjacent non-cancerous tissues were reduced in most clinical samples [24]. Consistent with these findings, our previous studies, miR-145 expression was downregulated in both clinical CRC samples and cell lines in comparison to normal colorectal tissues [24, 25]. Qing Liu et.al reported that serum expression level of miR-145 in CRC patients were significantly lower than that of healthy controls (*p* < 0.01). Furthermore, serum miR-145 expression showed a significant positive correlation with its expression in tumor tissue (*p* < 0.01) [26]. Based on our results, it appears that miR-145 decrease drug resistance by targeting the ABCC1 gene. Both mRNA and protein levels analysis of ABCC1 indicates that ABCC1 expression has significantly decreased following miR-145 overexpression. In silico analysis (TargetScan, miRWalk and miRmap) recognized binding sites for miR-145-3p and miR-145-5p within the ABCC1 mRNA, and we detected concordant down regulation in ABCC1 mRNA and protein levels after miR-145 restoration, following with decreased in cell viability and increased in apoptosis. While these data support a correlation between miR-145 and ABCC1 in oxaliplatin-resistant CRC cells. Therefore, our results suggest that ABCC1 is possibly a real target of miR-145 in our model. Gao et al. reported that miR-145 directly binds to the 3′-UTR of ABCC1 mRNA, leading to downregulation of its expression. They further demonstrated that miR-145 enhances the sensitivity of breast cancer cells to doxorubicin, supporting its role in overcoming drug resistance [21]. Unlike most ABC transporters, which are mostly found on apical membranes, ABCC1 is predominantly found in the basolateral membrane of polarized cells. These unique characteristics are supposed to confer different functional properties. Recent studies suggest that multidrug resistance is frequently associated with the upregulation of membrane transporters such as P-glycoprotein (MDR1) and multi-drug proteins (MRPs). These proteins neutralize the effects of the chemotherapy approach mainly by drug efflux [27]. However, drug resistance in CRC is multifactorial, and miR-145 represents just one of many potential therapeutic targets. In addition to miRNAs, lncRNAs also have vital roles in controlling key cancer-driving pathways, such as the JAK/STAT signaling pathway, which significantly influences colorectal cancer (CRC) progression and drug resistance, highlighting their potential as therapeutic targets [28]. In this study, we have evaluated MDR1mRNA expression levels as a representative example of dysregulated multi-DR genes. After miR-145 restoration (and other treatment groups) MDR1 expression was significantly lowered. However, ABCC1 was significantly upregulated in response to oxaliplatin monotherapy, and then lowered after miR-145 restoration, proposing that chemoresistance may be regulated via ABCC1 rather than MDR1 in the oxaliplatin resistant SW-480 cells. The results of cell death analysis showed that oxaliplatin monotherapy induced minimal apoptosis, whereas a significant increase in apoptotic cell death was observed in the group treated with the combination of miR-145 and oxaliplatin. Therefore, the observed alterations in CASP-3, CASP-8, CASP-9, and Bcl-2 mRNA expression most likely were related with apoptosis induction, characterized by the upregulation of pro-apoptotic genes and the downregulation of the anti-apoptotic gene (Bcl-2). Even though Bcl-2 expression was significantly down regulating via miR-145 restoration, its level did not further reduce upon addition of oxaliplatin. This indicates that miR-145 already achieves near-maximal inhibition of this anti-apoptotic gene. Oxaliplatin mainly acts via excerting DNA damage, which induce caspase-8 and − 9, which in the end promote caspase-3–mediated execution of apoptosis. Therefore, even though Bcl-2 mRNA levels remain similar between the miR-145 and oxa + miR-145 groups, apoptosis considerably increased in the combination treatment group due to the synergistic activation of downstream caspase pathways. Although these genes are not direct targets of miR-145, restoring the anti-cancer effects of oxaliplatin by miR-145 overexpression, may be explain the existing changes which indirectly impacts apoptotic signaling pathways. It was determined that high expression of miR-145 significantly reduced proliferation, migration, and apoptosis in gastric cancer cell line. These effects are caused by suppressing c-Myc, K-Ras, Bcl-2, and MMP-9 at the same time as boosting CASP3, CASP9, and Bax expression [29]. MTT results indicated that miR-145 in combination with oxaliplatin had a strong inhibitory effect on the growth and viability of oxaliplatin-resistant cells. Specifically, a ~ 40% reduction was observed in the survival rate as compared to oxaliplatin monotherapy. As support for the hypothesis, K-Ras mRNA expression level considerably decreased in the combination treatment group in comparison with those receiving oxaliplatin or miR-145 treatment alone. These findings with previous researches indicating that miR-145 hasas a tumor suppressor role because it negatively regulates multiple oncogenes such as Myc, K-Ras, IRS-1, ERK5. Moreover, miR-145 negatively regulates junctional cell adhesion molecule (JAM-A), fascin, and MUC1, leading to suppressed growth and differentiation in cancer stem cells. miR-145 also inhibits colon cancer cells’ proliferation and sensitizes them to 5-fluorouracil by targeting oncogenic FLI1 [30].The wound-healing (scratch) assay used to evaluate cell migration showed an inhibitory effect on the motility of miR-145 treated cells compared with cells treated with chemotherapy agent alone. In support of that, the evaluation of mRNA level indicates that expression of MMP-13 in SW-480 cell line reduces after combination treatment in comparison to single treatments. Bioinformatic analysis of ABCC1 in colorectal cancer revealed that the gene is upregulated at both the mRNA and protein levels. In line with these results, qRT-PCR analysis demonstrated a significant reduction in ABCC-1 mRNAs in the miR-145 transfected group compare with the control group. Similarly, ABCC1 protein level was reduced in the combination treatment group versus the blank control group. Based on a previous study, ABCC1 expression was significantly upregulated in colorectal cancer cells [31] and it was detected in most of human colorectal carcinoma cell lines [32]. Recent findings show that ABCC1 mRNA levels are significantly elevated in tumor tissue from patients with locally advanced and metastatic colorectal carcinoma compared with control tissues [33]. Moreover, ABCC1 mRNA levels were found to be increased in well-differentiated colon adenocarcinomas, suggesting that ABCC1 may be involved in the progression of colorectal cancer [34]. In conclusion, our findings focus the promising potential of miR-145 to help controlling oxaliplatin resistance in CRC cells. While earlier researches exhibited that miR-145 acts as a tumor suppressor via regulation individual oncogenes, our research indicating that restoring miR-145 may also control the ABCC1 transporter, resistant cells to oxaliplatin.

## Supplementary Information

Supplementary Material 1.

Supplementary Material 2.

Supplementary Material 3.

## Data Availability

All data supporting the findings of this study are available within the paper.

## Abbreviations

ABCC1: ATP Binding cassette C1
BSA: Bovine serum albumin
CASP − 8: Caspases-8
CASP − 9: Caspases-9
CASP-3: Caspases-3
cDNA: Complementary DNA
CPTAC: Clinical proteomic tumor consortium
CRC: Colorectal cancer
DMSO: Dimethyl sulfoxide
DNA: Deoxyribonucleic acid
ERK5: Extracellular signal-regulated kinase 5
FBS: Fetal bovine serum
GFP: Green fluorescent protein
JAM-A: Junctional cell adhesion molecule
LB agar: Luria broth agar
MDR1: Multidrug resistance 1
miRNAs: micrornas
MRPs: Multidrug resistance protein 1
MUC1: Mucin 1, cell surface associated
OD: Optical density
pCMV: Porcine cytomegalovirus
PCR: Polymerase chain reaction
qrt-PCR: quantitative real-time PCR
RNA: Ribonucleic acid
RPMI: Roswell Park Memorial Institute
SDS: Sodium dodecyl sulfate
UTR: Untranslated region
5-FU: 5-Fluorouracil
