# Supplementary material for: miR-145 restoration overcomes oxaliplatin resistance via ABCC1 in colorectal cancer
Source: J Egypt Natl Canc Inst. 2026 May 25;38:17. doi: 10.1186/s43046-026-00349-8 (PMC13313293; doi:10.1186/s43046-026-00349-8)
Supplement: Supplementary file 1 — Supplementary Material 1. [file 43046_2026_349_MOESM1_ESM.docx]

miR-145 Restoration Overcomes Oxaliplatin Resistance via ABCC1 in Colorectal Cancer

**Abstract**

**Background:** Drug resistance limits effectiveness of chemotherapy in colorectal cancer (CRC). Consequently, finding appropriate strategies for re-sensitizing chemo-resistant cells is crucial. Our present study aimed at taking advantage of chemotherapy and gene therapy by restoring microRNA-145 expression in oxaliplatin resistant CRC cells.

**Methods:** Bioinformatic analysis of clinical CRC datasets demonstrated significantly reduced miR‑145 expression in tumor tissues compared with normal samples. Oxaliplatin‑resistant SW‑480 cells were generated and transfected with a pCMV‑miR‑145 expression vector. The MTT assay was used to assess cell viability following miR‑145 restoration, oxaliplatin treatment, or their combination. Apoptosis was measured via flow cytometry. Gene expression levels of ABCC1, MDR1, K‑RAS, MMP‑13, Bcl‑2, CASP3, CASP8, and CASP9 were analyzed using qRT‑PCR, and ABCC1 protein expression was evaluated by western blotting. Cell migration was assessed using a wound‑healing assay.

**Results:** Co-treatment with miR-145 and Oxaliplatin significantly reduced cell viability, proliferation, and migration and increased apoptosis compared to the either treatments. Restoring miR‑145 expression downregulated reduced the drug‑resistance genes ABCC1 and MDR1, and reduced decreased expression of oncogenes including K‑RAS and Bcl‑2, while increasing expression of apoptosis‑related genes (CASP3, CASP8, and CASP9).

**Conclusion:** miR-145 restoration via decreasing the drug resistance biomarkers ABBCC1 and MDR1, along with other oncogenes like K-Ras and Bcl2, and increasing apoptosis conductors could sensitize oxaliplatin-resistant cells to chemotherapy. This proposes a novel and clinically translatable strategy to control drug resistance in CRC, finding new ways to increasing chemotherapeutic efficacy.

**Keywords**: Colorectal cancer; SW-480 cells; Oxaliplatin; Drug resistance; microRNA-145; Apoptosis, MDR1, ABCC1

**Introduction**

One of the most prevalent cancers of the digestive system, colorectal cancer (CRC) is the third most common cause of cancer-related death in the world [1]. Drug resistance is a main challenge in colorectal cancer therapy, with mechanisms including changing in non-coding RNAs leading to significantly to therapeutic failure. Recent research have highlighted the role of small RNAs, including microRNAs (miRNAs) and tRNA-derived small RNAs (tsRNAs), in controlling drug resistance via modulating key signaling pathways and drug transporters [2, 3]. Despite significant advances in CRC diagnosis and treatment , survival rates in late-stage disease are poor due to drug resistance and chemotherapy failures [4, 5]. Oxaliplatin is a platinum‑based chemotherapeutic agent that usually combined with other chemotherapy drugs (such as Irinotecan, 5-FU, and leucovorin), as standard treatment for stage II and stage III CRC [6, 7]. However, the problem is acquired drug resistance limits the effectiveness of this agent as a cancer treatment. The lack of knowledge of the mechanisms responsible for drug resistance hampers efforts to develop effective therapeutics [8].

Recently, combination of chemotherapy and gene-based therapy have been proposed as a cancer treatment [9]. A recent strategy in this area is microRNA replacement therapy, which introduces tumor-suppressor miRNAs into malignant cells [10].microRNAs (miRNAs) are categorized as oncogenic and tumor suppressor microRNAs [11]. They are important regulators of several cellular processes, such as cell proliferation, apoptosis, and differentiation [12]. Dysregulation of miRNA expression is found in a wide range of human cancers. A decrease in the expression level of tumor suppressor miRNAs can result in uncontrolled growth via upregulation of oncogenes and anti-apoptotic genes [13, 14]. The miR-145 is significantly down-regulated in several types of cancer, including colorectal cancer, kidney, esophageal, bladder, and breast cancer [11, 15]. MiR-145 regulates various essential cellular events, including proliferation, differentiation, apoptosis, invasion and metastasis [11, 16]. Specifically, miR-145 can increase the sensitivity of cancer cells to therapeutic agents by targeting important resistance-associated genes, such as Bcl-2, and ABCC1 [8, 15, 17, 18]. Although miR-145 functions as a tumor suppressor, its role in reversing oxaliplatin resistance in colorectal cancer especially through regulation of drug-efflux transporters like ABCC1 remains unclear. We hypothesized that restoring miR-145 in oxaliplatin-resistant CRC cells would sensitize them to treatment by suppressing ABCC1 and other oncogenic pathways.

**Methods**

Cell culture

The human colorectal cancer cell line (SW-480) was purchased from the Pasteur Institute, Iran and cells were cultured in Roswell Park Memorial Institute (RPMI)-1640 medium containing 10% fetal bovine serum (Gibco Laboratories, Grand Island, NY), 100 IU/ml penicillin and 100 μg/ml streptomycin. Cultures were incubated at a 37°C incubator (Memmert, Schwabach, Germany) with 5% CO_2_ and a humidified atmosphere. In all experimental procedures, cells were used in a logarithmic growth phase. The oxaliplatin-resistant SW-480 cells were generated by exposing the parental cells to increasing concentrations of oxaliplatin of . The initial dose for this purpose was 0.5 µM (cells were treated with this concentration for 60 days then concentration gradually increase 0.1 µM every week up to 2µM) that reached 2µM after 5 months.

Bioinformatics analysis

The Cancer Genome Atlas (TCGA) dataset was used to evaluate MiR-145 expression levels in 251 colon adenocarcinomas and 7 normal tissues. The data were analyzed by R using limma, and GEOquery package. In the context of clinical information, miR-145 expression levels were determined in colorectal primary tumors, individual cancer stages, and nodal metastases. A P-value of <0.05 compared to normal samples was considered statistically significant. Potential targets of miR-145 were predicted using the online prediction tools, Target scan, miRWalk and miRmap.

Plasmid vector preparation

A plasmid vector encoding miR-145 (Figure 1) was obtained from OriGene Company. Empty pCMV vector was used as the negative control. Both pCMV-miR-145 (vec+) and empty pCMV (vec−) vectors contained G-418 (Geneticin) resistance, as well as GFP coding sequence for selection and screening purposes. *Escherichia coli* (TOP10 strain)

was transformed as a host for plasmid proliferation by the method described before [19]. Briefly, *E. coli* bacteria were cultured in LB broth medium. When the optical density (OD600) reached 0.9, bacteria were harvested by centrifugation at 1000 g. Afterwards, competent cells were prepared by treating the bacteria with an ice-cold solution of 80 mM CaCl_2_ and 20 mM MgCl_2_. The transformation was done by 10 min of vibrating at 1000 rpm at 37°C using an “Eppendorf thermomixer C”. Transformed cells were plated on LB agar plates containing 30μg/ml kanamycin were used for screening of positive colonies. Positive colonies expressing GFP were selected, and plasmid Maxi Kit (QIAGEN, 12162) was utilized for plasmid DNA extraction from the confirmed colonies.

Transfection of SW-480 cells

Transfection was performed by electroporatino (Gene Pulser Xcell^(TM)^, Bio-rad, USA) and a quantity of 5×10^5^ oxaliplatin resistant SW-480cells were resuspended in electroporation buffer. Electcroporation buffer contained HEPES21 mM, NaCl 137mM, KCL 5mM, Na_2_HPO_4_, 7H_2_O 0.7mM and Dextrose 6mM. A single 200 V pulse, with a duration of 25ms, was utilized for transfection of 10μg of the pCMV-miR-145 vector and the same protocol used for blank pCMV vector (negative control). After 24h, cells were monitored by a live cell imaging system (Cytation 5, Biotek, Winooski, VT) for GFP expression and successful transfection (Figure 2A).

Moreover, restoration of miR-145 expression was confirmed by quantitative real-time PCR (qRT-PCR). For this purpose, total RNA was extracted using the RiboEx reagent (GeneAll biotechnology, Seoul, Korea) according to the manufacturer’s instructions. The quality and concentration of the extracted RNA was assessed by gel electrophoresis and NanoDrop OneCspectrophotometer (Thermo Scientific, USZ). The cDNA for miRNA quantification was synthesized from 10ng of the total RNA using the universal cDNA synthesis Kit (Exiqon, Vedbæk, Denmark). The qRT-PCR was performed by SYBR Green method and miR-145 specific primers (Exiqon, Denmark) using LightCycler 96 equipment (Roche Diagnostics, Mannheim, Germany). The two-step PCR comprised 45 cycles of 96°C for 10secs and 60°C for 1min. The expression of miR-145 was assessed and compared with the control group (transfected with an empty vector) using the (2^−ΔΔCT^) formula. Normalization was performed using U6.

Cell viability assay

The MTT assay was employed for the assessment of cell cytotoxicity following the individual miR-145 restoration, oxaliplatin treatment and combination of both oxaliplatin treatment and miR-145 overexpression. MTT assay was performed by seeding 3×10^3^ oxaliplatin resistant cells in 96 well culture plates and transfecting the cells with miR-145. Since continuous exposure to oxaliplatin was required to maintain the chemoresistant phenotype, cells have to be treated with oxaliplatin regularly in order to simulate chemo-resistance. Nevertheless, to decrease the electroporation-induced cellular stress,, oxaliplatin treatment was delayed for 48 hours after transfection to give cells time to recover. Afterwards, cells were treated with the same concentration of this drug (2ng/ml). After 24h of oxaliplatin treatment, the effect of miR-145 restoration on the viability of the oxaliplatin resistant cells was assessed by 3-(4, 5-dimethylthiazol-2-yl)-2, 5-diphenyl tetrazolium bromide (MTT) assay. MTT powder (Sigma, Taufkirchen, Germany) was used as a reagent at a concentration of 0.02 mg/ml (in RPMI-1640 medium containing 10% FBS). Then, cells were placed in a 37°C incubator. After 4 hours, the medium was replaced with 100μl of dimethyl sulfoxide (DMSO) and 25μl of Sorenson's buffer (glycine 0.1 M, NaCl 0.1 M, pH: 10.5 with 0.1 NaOH pH 10.5). The plate was gently shaken and then incubated at 37°C for 30 minutes to fully dissolve. The plate was gently shaken and then incubated at 37 °C for 30 minutes to fully dissolve the formazan crystals.

Apoptosis assay

The annexin V/propidium iodide (PI) flow cytometry assay was used to measure apoptosis induction following individual miR-145 overexpression, oxaliplatin treatment and the combined treatment. A total of 1 × 10⁵ cells per well were seeded into 6-well plates and divided into four groups: (i) miR-145 overexpression, (ii) oxaliplatin treatment, (iii) combined miR-145 + oxaliplatin treatment, and (iv) negative control (vec−). Oxaliplatin-resistant cells transfected with empty vector (vec−) were considered as a negative control. A total of 1×10^5^ oxaliplatin resistant cells were seeded into 6-well plates, and divided into four groups: (i) miR-145 overexpression, (ii) oxaliplatin treatment, (iii) combined miR-145 + oxaliplatin treatment, and (iv) negative control (vec−). The subject was then treated with a chemotherapy agent and 24 hours later, the cells were trypsinized and centrifuged at 1,500g for 5 min. Next, the cells were stained with Annexin V and PI in accordance with the manufacturer's instructions (Roche). Briefly, the samples were incubated with 2ml of annexin V, 1ml of propidium iodide, and 100ml of binding buffer. Afterwards, the cells were incubated for 15min at room temperature under dark conditions. The samples were analyzed using a flow cytometry instrument (Macs Quant Analyser 10, Miltenyi Biotech, Germany). FlowJo software (Tree Star, San Carlos, CA) was used to assess the rate of apoptosis.

Wound healing assay

For evaluating cell migration in treated groups, we used a wound-healing (scratch) assay. Oxaliplatin exposure was temporarily suspended prior to transfection. Approximately 48 hours after electroporation, a chemotherapy agent was added at a dose of 1ng/ml. Oxaliplatin-resistant SW-480 cells were seeded in 24 well plates at a density of 25×10 3 cells, and once they achieved a confluency of 80%-90%, a yellow tip was used to generate wound gaps in cell monolayers. We monitored cell migration to fill the gaps after removing detached cells with fresh RPMI-1640 medium. Images of the wound area were captured at 0, 24, 48, and 72 hours to evaluate migration.

Assessment of mRNA expression by quantitative real-time PCR (qRT-PCR)

A qRT-PCR assay was employed to evaluate the expression levels of ABCC1, MDR1, K-RAS, MMP-13, Bcl-2, CAS3, -8 and CAS-9 in the treatment groups miR-145 overexpression, Oxaliplatin treatment, and their combination). A number of 5×10^5^ oxaliplatin-resistant cells were electroporated and allowed to recovered for 48 h in absence of a chemotherapy agent in a 25cm^2^ culture flask. After recovery, cells received the designated treatments, and 24h later, total RNA was extracted, using RiboEx (GeneAll, Seoul, Korea). Also, the quantity and quality of the extracted total RNA was evaluated by Nanodrop 2000 system (Thermo Fisher Scientific, Wilmington, DE, USA). Complementary DNA (cDNA) was synthesized using 1μg of RNA template by Revert Aid cDNA Synthesis kit (Thermo, USA). PCR conditions were an enzyme activation step of 95°C followed by 45 cycles of denaturing at 95°C for 10 secs, annealing at 60°C for 30 secs, and extension at 72°C for 20 secs. Real-time PCR was performed using SYBR Green PCR Pre-Mix (Ampliqon, Denmark) and GAPDH was used as the housekeeping gene. The expression levels were quantified using light cycler systems 96 (Roche, Germany) equipment. Relative mRNA expression levels were calculated using the 2⁻ΔΔCt (Livak) method. The primer sets used for the quantification of target genes are provided in Table 1 with more details.

Western blot analysis

Western blotting was performed to determine whether changes in ABCC1 protein levels is related to miR-145 expression,. For this purpose, 1×10^5^oxaliplatin resistant SW-480 cells were transfected and seeded into a 6-well culture plate. Oxaliplatin treatment was continued 48 hours following electroporation. After that, cells were detached and lysed in RIPA buffer (Santa Cruz Biotech, USA) for protein extraction. A Bradford assay was done by using a NanoDrop 2000c spectrophotometer (Thermo Fisher Scientific, USA) to quantify the protein concentration, and a 12.5% SDS polyacrylamide gel was used to separate 25μg of the protein lysate. The protein bands were transferred onto polyvinylidene difluoride membranes (Millipore, USA) using a semi-dry electroblotting system (Bio-Rad Laboratories, USA). Membranes were blocked with 3% bovine serum albumin (BSA), and subsequently membranes were incubated with primary mouse monoclonal antibodies against ABCC1 (1:1000, IU2H10, Novus Biologicals) and β-actin (1:1000, ab8227, Abcam) as the loading control. After washing, membranes were incubated with horseradish peroxidase-linked goat anti-mouse antibody (1:3000, Abcam) for one hour at room temperature. To visualize bands, a chemiluminescence detection kit (Roche Diagnostics GmbH, Germany) was used with an automatic imager.

Statistical analysis

The statistical analysis was performed using GraphPad Prism (version 6. 0, San Diego, CA). All data were stated as means ± SEM (standard error mean). All experiments were performed independently at least three times. Student's t and analysis of variance (ANOVA) tests were exploited to determine the statistical significance of differences and p values< 0. 05 were considered significant.

**Results**

hsa-MiR-145 prevented the proliferation of oxaliplatin resistant SW-480 cells

In colon adenocarcinomas samples, miR-145 expression was significantly decreased in primary tumors compared to normal tissues (Figure 2A). Additionally, miR-145 expression levels were significantly lower in stages (I–IV) as well as N0-N2 nodal metastasis compared to normal tissues (Figures 2B, 2C). Although miR-145 was considerably down regulate in all tumor nodal subgroups (N0–N2) compared with normal tissues (*** *P < 0.001*), we did not observe significant statistically differences among N0, N1, and N2. This shows that miR-145 suppression happens early and remains relatively stable across nodal stages progresses.

Transfection of the oxaliplatin resistant CRC cells

The microRNA expression vector backbone contains a GFP reporter sequence. Thus, the successful transfection of the CRC cells was confirmed by the expression of GFP as shown (Figure 3A). Additionally, miRNA analysis revealed a 14.2fold increase in miR-145 expression in cells transfected the pCMV-miR-145 vector compared with the blank control (Figure 3B).

MiR-145 restoration leads to sensitization of the oxaliplatin resistant colorectal cancer cells

As a result of overexpression of miR-145, the effects of the expression on drug sensitivity have been detected using the MTT assay. As shown in Figure 4A, the proliferation rate of cells treated with the miR-145 vector and Oxaliplatin combination significantly decreased compared with the control group (*p* < 0.0001), cells treated with Oxaliplatin alone (*p* < 0.0001) and cells treated with miR-145 alone (*p* = 0.0035). Correspondingly, according to the results of qRT-PCR, the expression level of MDR-1, ABCC1 and K-RAS in the group treated with the combination of miR-145 and Oxaliplatin had significantly decreased in comparison with the cells treated with oxaliplatin alone (*p* < 0.0001) (Figure 4B-4D). To evaluate the miR-145's impact on the apoptotic rate of oxaliplatin-resistant SW-480 cells, an annexin V/PI flow cytometry assay was employed. Flow cytometry results have indicated that the percentage of late apoptosis in the group treated with combined miR-145 and Oxaliplatin was markedly higher than in the only oxaliplatin treated group (1.682% vs. 18.11%). Together, these studies showed that miR-145 overexpression significantly reduced the resistance of Oxaliplatin resistant SW-480 cells to oxaliplatin (Figure 4E). These results were supported with the mRNA expression levels of CAS3, -8, -9, and Bcl-2 genes. A significant down regulation in Bcl-2 mRNA expression was observed in the group treated with a combination of miR-145 and Oxaliplatin compared with the control group (*p* = 0.0001) and the group treated with only Oxaliplatin (*p* = 0.0001). However, there was no difference compared with the group treated with only miR-145 (*p* = 0.237) (Figure 4F). There was a significant increase in mRNA expression of CAS-3, CAS-8, and CAS-9 in the combination group in comparison to the other groups. Specifically, CAS-3 mRNA expression was considerably up regulated in the group treated with a combination of miR-145 and oxaliplatin in comparison to the control group (*p* < 0.0001), the group treated with only miR-145 (*p* = 0.00081), and the group treated with only oxaliplatin (*p* = 0.00081).

Additionally, the expression level of CAS-8 and CAS-9 mRNA in the combination of miR-145 and oxaliplatin were significantly increased in comparison to the control group (*p* < 0.0001) and the group treated with only oxaliplatin (*p* < 0.0001). However,there was no significant change in comparison to the group treated with only miR-145 (*p* = 0.451 and *p* = 0.279, respectively) (Figures 4G–4I). To evaluate the inhibitory effect of miR-145 on the migration of oxaliplatin-resistant CRC cells, a wound-healing (scratch) assay was employed. Oxaliplatin- resistant SW-480 cells treated with combined oxaliplatin and miR-145 were compared with cells exposing empty vectors (negative control) and oxaliplatin. Compared to the controls, cells overexpressing miR-145 showed a significant decrease in migration in 24, 48, and 72 h (Figure 4J). Consequently, qRT-PCR analysis showed significant reduction in MMP-13 mRNA expression levels in the combined miR-145-oxaliplatin treatment compared to the control group (*p* < 0.0001) and the group exposed to oxaliplatin alone (Figure 4K). It was not significantly different between groups treated with miR-145 alone versus those treated with the combination (*p* = 0.91).

RNA and protein expression analysis of drug resistance gene

A bioinformatics target prediction analysis indicated that miR-145-3p and -5p may directly target ABCC1 mRNA, with prediction miRmap scores of 21.41 and 50.81, respectively (Figure 5A). A significant reduction in ABCC-1 mRNA and protein expression occurred after miR-145 transfection in comparison to the control group (Figure 5B and 5C). Further bioinformatics analysis of ABCC1 levels in the clinical colorectal samples of the COAD project (TCGA dataset) revealed ABCC1 overexpression in primary tumors (Figure 6A), across stage I–IV (Figure 6B), and in nodal metastasis groups (N0-N2) colorectal tumor samples (Figure 6C). Additionally, based on the data of the Cancer Institute's Clinical Proteomic Tumor Consortium (CPTAC), the protein expression level of ABCC1 increased at a significant rate in colorectal cancer samples (Figure 6D).

**Discussion**

Globally, CRC is one of the most prevalent malignancies of the digestive system and remains a major cause of cancer-related mortality. Although there has been significant progress in the diagnosis and treatment of CRC, there still exists a low survival rate of cancer patients. In this study, we demonstrate that miR-145 sensitizes oxaliplatin-resistant SW-480 cells to oxaliplatin. There have been several studies that suggest miR-145 could plays a tumor-suppressive role and may reduce chemoresistance in various cancers [8, 20]. Oxaliplatin is a widely used chemotherapy agent for colorectal cancer and several other malignancies, either as monotherapy or in combination with other therapeutics such as Fluorouracil [21]. However, acquired drug resistance is one of the most common limiting factors in the treatment of CRC. To overcome this limitation, combinational strategies such as chemo/gene silencing or miRNA-based approaches have emerged to improve therapeutic effectiveness and remedy the existing disadvantages [22]. In our study, bioinformatics analysis revealed that miR-145 was considerably downregulated in colorectal cancer tissues compared with normal tissues. It has been reported that miR-145 has been reduced in precancerous colorectal lesions as well as in different stages of colorectal cancer tissue samples compared to normal colorectal mucosa [23]. Several studies have suggested that the miRNA expression profiles of 31 pairs CRC tissues and adjacent non-cancerous tissues were reduced in most clinical samples [24]. Consistent with these findings, our previous studies, miR-145 expression was downregulated in both clinical CRC samples and cell lines in comparison to normal colorectal tissues [24, 25]. Qing Liu et.al reported that serum expression level of miR-145 in CRC patients were significantly lower than that of healthy controls (*p* < 0.01). Furthermore, serum miR-145 expression showed a significant positive correlation with its expression in tumor tissue (*p* < 0.01)[26]. Based on our results, it appears that miR-145 decrease drug resistance by targeting the ABCC1 gene. Both mRNA and protein levels analysis of ABCC1 indicates that ABCC1 expression has significantly decreased following miR-145 overexpression. In silico analysis (TargetScan, miRWalk and miRmap) recognized binding sites for miR-145-3p and miR-145-5p within the ABCC1 mRNA, and we detected concordant down regulation in ABCC1 mRNA and protein levels after miR-145 restoration, following with decreased in cell viability and increased in apoptosis. While these data support a correlation between miR-145 and ABCC1 in oxaliplatin-resistant CRC cells, but we did not do luciferase 3′UTR reporter test or ABCC1 rescue experiments in this research. Therefore, our results suggest that ABCC1 is possibly a real target of miR-145 in our model. Gao et al. reported that miR-145 directly binds to the 3′-UTR of ABCC1 mRNA, leading to downregulation of its expression. They further demonstrated that miR-145 enhances the sensitivity of breast cancer cells to doxorubicin, supporting its role in overcoming drug resistance [21]. Unlike most ABC transporters, which are mostly found on apical membranes, ABCC1 is predominantly found in the basolateral membrane of polarized cells. These unique characteristics are supposed to confer different functional properties. Gao et al. demonstrated that miR-145 directly binds to the 3' UTR of ABCC1. In addition, they demonstrated that miR-145 induces the sensitivity of breast cancer cells to doxorubicin drugs, supporting its role in overcoming drug resistance [21]. Recent studies suggest that multidrug resistance is frequently associated with the upregulation of membrane transporters such as P-glycoprotein (MDR1) and multi-drug proteins (MRPs). These proteins neutralize the effects of the chemotherapy approach mainly by drug efflux [27]. However, drug resistance in CRC is multifactorial, and miR-145 represents just one of many potential therapeutic targets. In addition to miRNAs, lncRNAs also have vital roles in controlling key cancer-driving pathways, such as the JAK/STAT signaling pathway, which significantly influences colorectal cancer (CRC) progression and drug resistance, highlighting their potential as therapeutic targets [28]. In this study, we have evaluated MDR1mRNA expression levels as a representative example of dysregulated multi-DR genes. After miR-145 restoration in oxaliplatin resistant cells, MDR1 expression was not significantly lowered, proposing that miR-145 mainly regulate chemoresistance via other targets like ABCC1 rather than MDR1. The results of cell death analysis showed that oxaliplatin monotherapy induced minimal apoptosis, whereas a significant increase in apoptotic cell death was observed in the group treated with the combination of miR-145 and oxaliplatin. The result of cell death analysis demonstrated that the rate of apoptosis in oxaliplatin monotherapy was minimal, whereas a significant increase in apoptosis was observed in the combination of miR-145 and oxaliplatin treated group. Therefore, the observed alters in CAS-3, CAS-8, CAS-9, and Bcl-2 mRNA expression were related with apoptosis induction, characterized by the upregulation of pro-apoptotic genes and the downregulation of the anti-apoptotic gene (Bcl-2). Even though Bcl-2 expression was significantly down regulating via miR-145 restoration, its level did not further reduce upon addition of oxaliplatin. This indicates that miR-145 already achieves near-maximal inhibition of this anti-apoptotic gene. Oxaliplatin mainly acts via activating DNA damage, which induce caspase-8 and -9, which in the end promote caspase-3–mediated execution of apoptosis. Therefore, even though Bcl-2 mRNA levels remain similar between the miR-145 and oxa + miR-145 groups, apoptosis considerably increased in the combination treatment group due to the synergistic activation of downstream caspase pathways. Although these genes are not direct targets of miR-145, restoring the anti-cancer effects of oxaliplatin by miR-145 overexpression, may be explain the existing changes which indirectly impacts apoptotic signaling pathways. It was determined that high expression of miR-145 significantly reduced proliferation, migration, and apoptosis in gastric cancer cell line. These effects are caused by suppressing c-Myc, K-Ras, Bcl-2, and MMP-9 at the same time as boosting CASP3, CASP9, and Bax expression [29]. MTT results indicated that miR-145 in combination with oxaliplatin had a strong inhibitory effect on the growth and viability of oxaliplatin-resistant cells. Specifically, a ~40% reduction was observed in the survival rate as compared to oxaliplatin monotherapy. As support for the hypothesis, K-Ras mRNA expression level considerably decreased in the combination treatment group in comparison with those receiving oxaliplatin or miR-145 treatment alone. These findings with previous researches indicating that miR-145 hasas a tumor suppressor role because it negatively regulates multiple oncogenes such as Myc, K-Ras, IRS-1, ERK5. Moreover, miR-145 negatively regulates junctional cell adhesion molecule (JAM-A), fascin, and MUC1, leading to suppression and invasiveness of breast cancer cell motility. miR-145 also inhibits colon cancer cells’ proliferation and sensitizes them to 5-fluorouracil by targeting oncogenic FLI1 [30].The wound-healing (scratch) assay used to evaluate cell migration showed an inhibitory effect on the motility of miR-145 treated cells compared with cells treated with chemotherapy agent alone. In support of that, the evaluation of mRNA level indicates that expression of MMP-13 in SW-480 cell line reduces after combination treatment in comparison to single treatments. Bioinformatic analysis of ABCC1 in colorectal cancer revealed that the gene is upregulated at both the mRNA and protein levels. In line with these results, qRT-PCR analysis demonstrated a significant reduction in ABCC-1 mRNAs in the miR-145 transfected group compare with the control group. Similarly, ABCC1 protein level was reduced in the combination treatment group versus the blank control group. Based on a previous study, ABCC1 expression was significantly upregulated in colorectal cancer cells [31] and it was detected in most of human colorectal carcinoma cell lines [32]. Recent findings show that ABCC1 mRNA levels are significantly elevated in tumor tissue from patients with locally advanced and metastatic colorectal carcinoma compared with control tissues [33]. Moreover, ABCC1 mRNA levels were found to be increased in well-differentiated colon adenocarcinomas, suggesting that ABCC1 may be involved in the progression of colorectal cancer [34]. An important limitation of this research is that it uses only one colorectal cancer cell line (SW-480), that may restrict relation of finding to other CRC subtypes. In addition, we did not evaluate other genes or pathways which may impact oxaliplatin resistance. Although we evaluated the expression of some downstream genes which related to drug resistance, apoptosis and invasion (ABCC1, MDR1, KRAS, Bcl-2, CASP3, CASP8, CASP9 and MMP-13), but we did not do standard external validation test including luciferase 3′UTR reporter assay. Finally, we also did not evaluate the treatment long-term in vivo, we don’t know its side effects or if resistance might return. In conclusion, our findings focus the promising potential of miR-145 to help controlling oxaliplatin resistance in CRC cells. While earlier researches exhibited that miR-145 acts as a tumor suppressor via regulation individual oncogenes, our research indicating that restoring miR-145 may also control the ABCC1 transporter, which is important role in regulating multidrug resistance and re-sensitize resistant cells to oxaliplatin.

**Abbreviation list**

ABCC1: [ATP Binding Cassette C1](https://pubmed.ncbi.nlm.nih.gov/24224072/); BSA: Bovine serum albumin; CASP -8: Caspases-8; CASP -9: Caspases-9; CASP-3: Caspases-3; cDNA: Complementary DNA; CPTAC: Clinical Proteomic Tumor Consortium; CRC: Colorectal cancer; DMSO: dimethyl sulfoxide; DNA: Deoxyribonucleic Acid; ERK5: Extracellular signal-regulated kinase 5; FBS: Fetal Bovine Serum; GFP: Green fluorescent protein; JAM-A: junctional cell adhesion molecule; LB agar: [Luria Broth agar](https://asm.org/getattachment/5d82aa34-b514-4d85-8af3-aeabe6402874/LB-Luria-Agar-protocol-3031.pdf); MDR1: Multidrug resistance 1; miRNAs: micrornas; MRPs: Multidrug resistance protein 1; MUC1: Mucin 1, cell surface associated; OD: Optical density; pCMV: Porcine Cytomegalovirus; PCR: Polymerase chain reaction; qrt-PCR: quantitative real-time PCR; RNA: Ribonucleic Acid; RPMI: Roswell Park Memorial Institute; SDS: [Sodium Dodecyl Sulfate](https://www.thermofisher.com/order/catalog/product/28364); UTR: Untranslated region; 5-FU: 5-Fluorouracil.

**Funding**

The current study was financially supported by the Immunology Research Center, Tabriz University of Medical Sciences, Tabriz, Iran (grant number: 61907).

**Ethics approval**

The study was approved by the Ethical Committee of Tabriz University of Medical Sciences (IR. TBZMED. VCR. REC. 1397. 345).

**Consent to participate**

I would like to clarify that in this study, we did not involve any human participants. All research was conducted using established cell lines.

**References**

1 Siegel, R. L., Miller, K. D. and Jemal, A. (2020) Cancer statistics, 2020. CA: a cancer journal for clinicians. **70**, 7-30

2 Chen, Y., Liang, J., Chen, S., Lin, N., Xu, S., Miao, J., Zhang, J., Chen, C., Yuan, X., Xie, Z., Zhu, E., Cai, M., Wei, X., Hou, S. and Tang, H. (2024) Discovery of vitexin as a novel VDR agonist that mitigates the transition from chronic intestinal inflammation to colorectal cancer. Molecular Cancer. **23**, 196

3 Xu, R., Du, A., Deng, X., Du, W., Zhang, K., Li, J., Lu, Y., Wei, X., Yang, Q. and Tang, H. (2024) tsRNA-GlyGCC promotes colorectal cancer progression and 5-FU resistance by regulating SPIB. Journal of experimental & clinical cancer research : CR. **43**, 230

4 Dienstmann, R., Vermeulen, L., Guinney, J., Kopetz, S., Tejpar, S. and Tabernero, J. (2017) Consensus molecular subtypes and the evolution of precision medicine in colorectal cancer. Nature reviews cancer. **17**, 79-92

5 Kamran, S., Seyedrezazadeh, E., Shanehbandi, D., Asadi, M., Zafari, V., Shekari, N., Namvar, L. and Zarredar, H. (2021) Combination therapy with KRAS and P38α siRNA suppresses colorectal cancer growth and development in SW480 cell line. Journal of Gastrointestinal Cancer, 1-8

6 Chen, M. C., Hsu, H. H., Chu, Y. Y., Cheng, S. F., Shen, C. Y., Lin, Y. J., Chen, R. J., Viswanadha, V. P., Lin, Y. M. and Huang, C. Y. (2018) Lupeol alters ER stress‐signaling pathway by downregulating ABCG2 expression to induce Oxaliplatin‐resistant LoVo colorectal cancer cell apoptosis. Environmental toxicology. **33**, 587-593

7 Goldstein, D. A., Zeichner, S. B., Bartnik, C. M., Neustadter, E. and Flowers, C. R. (2016) Metastatic colorectal cancer: a systematic review of the value of current therapies. Clinical colorectal cancer. **15**, 1-6

8 Gao, M., Miao, L., Liu, M., Li, C., Yu, C., Yan, H., Yin, Y., Wang, Y., Qi, X. and Ren, J. (2016) miR-145 sensitizes breast cancer to doxorubicin by targeting multidrug resistance-associated protein-1. Oncotarget. **7**, 59714

9 To, K. K. (2013) MicroRNA: a prognostic biomarker and a possible druggable target for circumventing multidrug resistance in cancer chemotherapy. Journal of biomedical science. **20**, 1-19

10 Xia, C., Yang, Y., Kong, F., Kong, Q. and Shan, C. (2018) MiR-143-3p inhibits the proliferation, cell migration and invasion of human breast cancer cells by modulating the expression of MAPK7. Biochimie. **147**, 98-104

11 Sadeghiyeh, N., Sehati, N., Mansoori, B., Mohammadi, A., Shanehbandi, D., Khaze, V. and Baradaran, B. (2019) MicroRNA-145 replacement effect on growth and migration inhibition in lung cancer cell line. Biomedicine & Pharmacotherapy. **111**, 460-467

12 Cho, W. C., Wong, C. F., Li, K. P., Fong, A. H., Fung, K. Y. and Au, J. S. (2023) miR-145 as a potential biomarker and therapeutic target in patients with non-small cell lung cancer. International Journal of Molecular Sciences. **24**, 10022

13 Shanehbandi, D., Asadi, M., Seyedrezazadeh, E., Zafari, V., Shekari, N., Akbari, M., Rahbarnia, L. and Zarredar, H. (2023) microRNA-based biomarkers in lung cancer: recent advances and potential applications. Current Molecular Medicine. **23**, 648-667

14 Sachdeva, M. and Mo, Y.-Y. (2010) miR-145-mediated suppression of cell growth, invasion and metastasis. American journal of translational research. **2**, 170

15 Liu, R.-L., Dong, Y., Deng, Y.-Z., Wang, W.-J. and Li, W.-D. (2015) Tumor suppressor miR-145 reverses drug resistance by directly targeting DNA damage-related gene RAD18 in colorectal cancer. Tumor biology. **36**, 5011-5019

16 Cui, S. Y., Wang, R. and Chen, L. B. (2014) Micro RNA‐145: a potent tumour suppressor that regulates multiple cellular pathways. Journal of cellular and molecular medicine. **18**, 1913-1926

17 Kazmierczak, D., Jopek, K., Sterzynska, K., Ginter-Matuszewska, B., Nowicki, M., Rucinski, M. and Januchowski, R. (2020) The significance of MicroRNAs expression in regulation of extracellular matrix and other drug resistant genes in drug resistant ovarian cancer cell lines. International journal of molecular sciences. **21**, 2619

18 Zhang, Y. and Wang, J. (2017) MicroRNAs are important regulators of drug resistance in colorectal cancer. Biological chemistry. **398**, 929-938

19 Shanehbandi, D., Saei, A. A., Zarredar, H. and Barzegari, A. (2013) Vibration and glycerol-mediated plasmid DNA transformation for Escherichia coli. FEMS microbiology letters. **348**, 74-78

20 Fu, Q., Cheng, J., Zhang, J., Zhang, Y., Chen, X., Xie, J. and Luo, S. (2017) miR-145 inhibits drug resistance to Oxaliplatin in colorectal cancer cells through regulating G protein coupled receptor 98. Zhonghua wei chang wai ke za zhi= Chinese journal of gastrointestinal surgery. **20**, 566-570

21 Gao, R., Fang, C., Xu, J., Tan, H., Li, P. and Ma, L. (2019) LncRNA CACS15 contributes to oxaliplatin resistance in colorectal cancer by positively regulating ABCC1 through sponging miR-145. Archives of biochemistry and biophysics. **663**, 183-191

22 Yang, F., Xie, Y.-Q., Tang, S.-Q., Wu, X.-B. and Zhu, H.-Y. (2015) miR-143 regulates proliferation and apoptosis of colorectal cancer cells and exhibits altered expression in colorectal cancer tissue. International journal of clinical and experimental medicine. **8**, 15308

23 Michael, M. Z., O'Connor, S. M., van Holst Pellekaan, N. G., Young, G. P. and James, R. J. (2003) Reduced Accumulation of Specific MicroRNAs in Colorectal Neoplasia11Note: Susan M. O'Connor and Nicholas G. van Holst Pellekaan contributed equally to this work. Molecular cancer research. **1**, 882-891

24 Xu, X.-H., Wu, X.-B., Wu, S.-B., Liu, H.-B., Chen, R. and Li, Y. (2014) Identification of miRNAs differentially expressed in clinical stages of human colorectal carcinoma—An investigation in Guangzhou, China. PloS one. **9**, e94060

25 Li, S., Wu, X., Xu, Y., Wu, S., Li, Z., Chen, R., Huang, N., Zhu, Z. and Xu, X. (2016) miR-145 suppresses colorectal cancer cell migration and invasion by targeting an ETS-related gene. Oncology reports. **36**, 1917-1926

26 Liu, Q., Yang, W., Luo, Y., Hu, S. and Zhu, L. (2018) Correlation between miR-21 and miR-145 and the incidence and prognosis of colorectal cancer. JBUON. **23**, 29-35

27 Cao, D., Qin, S., Mu, Y. and Zhong, M. (2017) The role of MRP1 in the multidrug resistance of colorectal cancer. Oncology letters. **13**, 2471-2476

28 Ghasemian, A., Omear, H. A., Mansoori, Y., Mansouri, P., Deng, X., Darbeheshti, F., Zarenezhad, E., Kohansal, M., Pezeshki, B., Wang, Z. and Tang, H. (2023) Long non-coding RNAs and JAK/STAT signaling pathway regulation in colorectal cancer development. Front Genet. **14**, 1297093

29 Wang, J., Sun, Z., Yan, S. and Gao, F. (2019) Effect of miR‑145 on gastric cancer cells. Molecular Medicine Reports. **19**, 3403-3410

30 Yu, Y., Nangia-Makker, P., Farhana, L., Rajendra, S. G., Levi, E. and Majumdar, A. P. (2015) miR-21 and miR-145 cooperation in regulation of colon cancer stem cells. Molecular cancer. **14**, 1-11

31 Chen, M., Li, D., Gong, N., Wu, H., Su, C., Xie, C., Xiang, H., Lin, C. and Li, X. (2017) miR-133b down-regulates ABCC1 and enhances the sensitivity of CRC to anti-tumor drugs. Oncotarget. **8**, 52983

32 Ku, J.-L., Shin, Y.-K., Kim, D.-W., Kim, K.-H., Choi, J.-S., Hong, S.-H., Jeon, Y.-K., Kim, S.-H., Kim, H.-S. and Park, J.-H. (2010) Establishment and characterization of 13 human colorectal carcinoma cell lines: mutations of genes and expressions of drug-sensitivity genes and cancer stem cell markers. Carcinogenesis. **31**, 1003-1009

33 Hinoshita, E., Uchiumi, T., Taguchi, K.-i., Kinukawa, N., Tsuneyoshi, M., Maehara, Y., Sugimachi, K. and Kuwano, M. (2000) Increased expression of an ATP-binding cassette superfamily transporter, multidrug resistance protein 2, in human colorectal carcinomas. Clinical cancer research. **6**, 2401-2407

34 Kunická, T. and Souček, P. (2014) Importance of ABCC1 for cancer therapy and prognosis. Drug metabolism reviews. **46**, 325-342


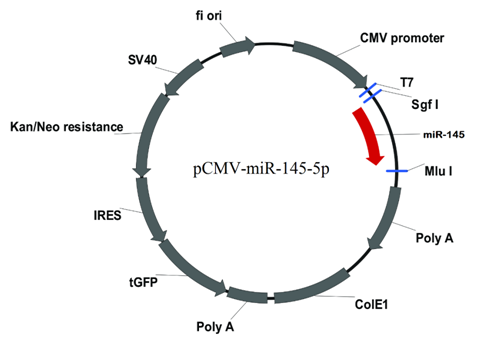


**Fig.1.** **Schematic representation of the pCMV-miR-145 expression vector.**
The map illustrates the main functional elements of the pCMV-miR-145 construct used to overexpress miR-145 in colorectal cancer cells.


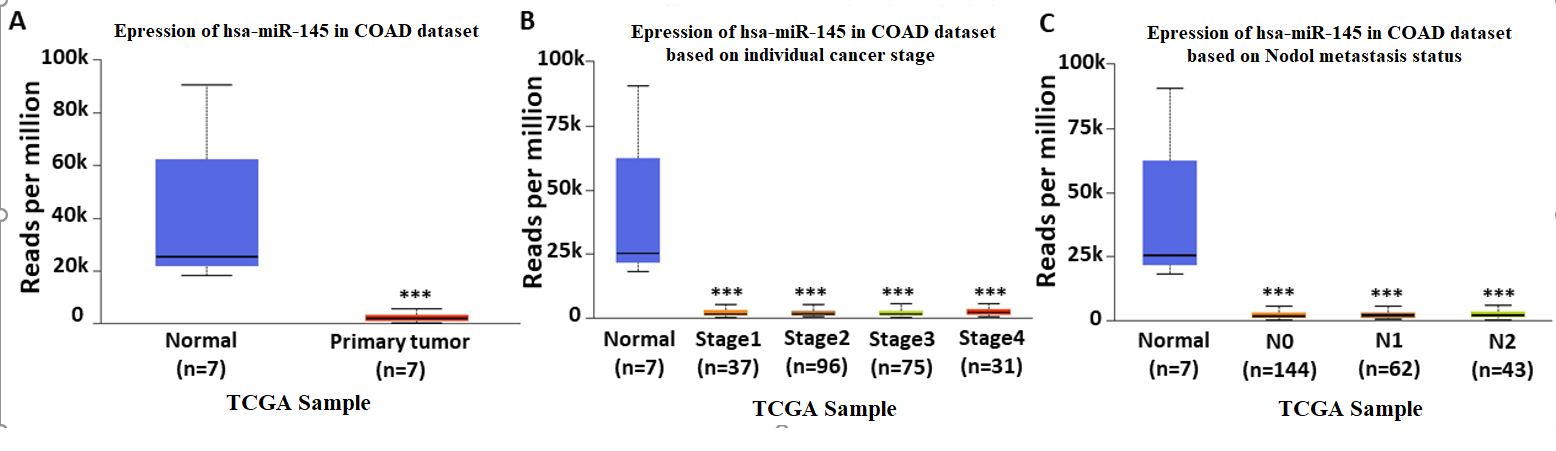


**Fig.2. miR-145 expression reduced in colon adenocarcinoma.** Expression data from the TCGA-COAD project show that miR-145 levels are significantly decreased in primary tumor tissues compared with normal colon tissues (A). miR-145 expression is consistently reduced across tumor stages I–IV (B) and across nodal status groups N0–N3 compared with normal tissues (C). ***P < 0.001 versus normal tissues.


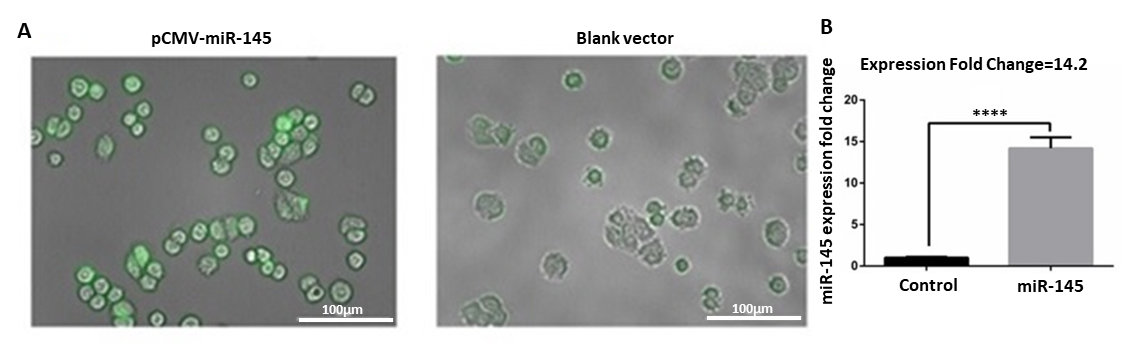


**Fig.3. pCMV-miR-145 induces miR-145 expression in oxaliplatin-resistant colorectal cancer cells.** GFP expression in oxaliplatin-resistant cells transfected with pCMV-miR-145 (left) or empty vector (right) confirms successful transfection (A). miR-145 expression was increased more than 14-fold in cells transfected with pCMV-miR-145 compared with cells transfected with the empty vector (P < 0.0001) (B).


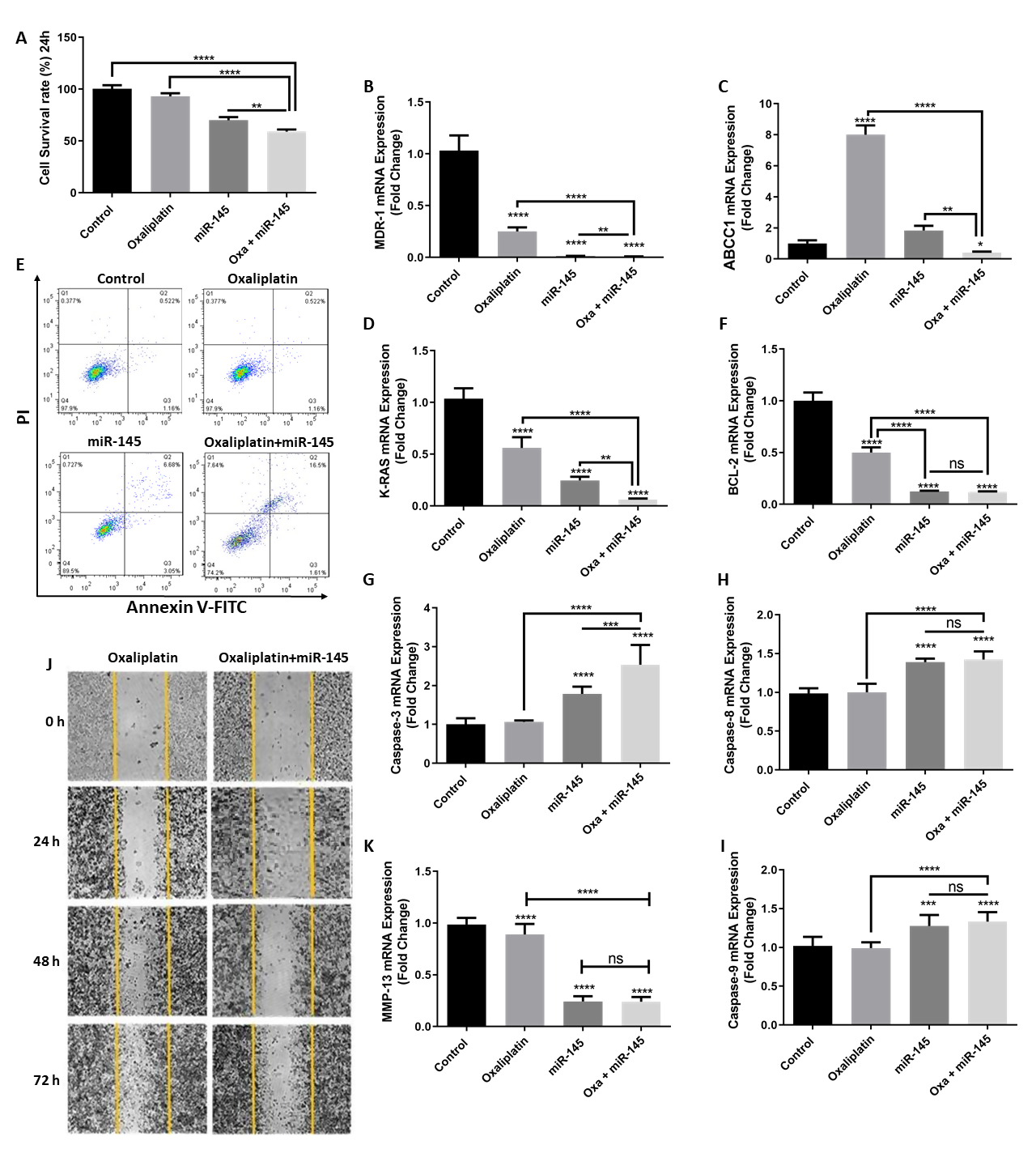


**Fig.4. Combined oxaliplatin and miR-145 treatment induces cell death and suppresses migration in oxaliplatin-resistant SW-480 cells.** MTT assay showed a significant decrease in cell viability in the group treated with both pCMV-miR-145 and oxaliplatin compared with oxaliplatin alone (****: P.V <0.0001) (A). MDR1 mRNA levels were significantly reduced in cells treated with miR-145 alone and in those receiving the combination treatment compared with oxaliplatin-only cells (****: P.V <0.0001) (B). ABCC1 mRNA expression was decreased in cells treated with both oxaliplatin and miR-145 compared with control and single-agent treatments (C). K-RAS mRNA expression was reduced in the combination group compared with oxaliplatin-alone cells (****: P.V <0.0001) (D).

The combination of Oxaliplatin and miR-145 overexpression enhances the apoptosis rate in oxaliplatin-resistant SW-480 cells. Late apoptosis increased in the test group with both exogenous miR-145 and Oxaliplatin (E). In cells receiving both Oxaliplatin and miR-145 Bcl2 mRNA expression decreased, whereas, mRNA level of CAS3, CAS8 and CAS9 were significantly increased compared with control group (****: P.V <0.0001, ****: P.V <0.0001, ****: P.V<0.0001)( F-I). Wound healing assay showed miR-145 restoration harnessed the migratory potential of Oxaliplatin resistant SW-480 cells (J). MMP-13 mRNA levels were significantly reduced in cells treated with miR-145 alone and in the combination, group compared with oxaliplatin-only cells (K). ****P < 0.0001.


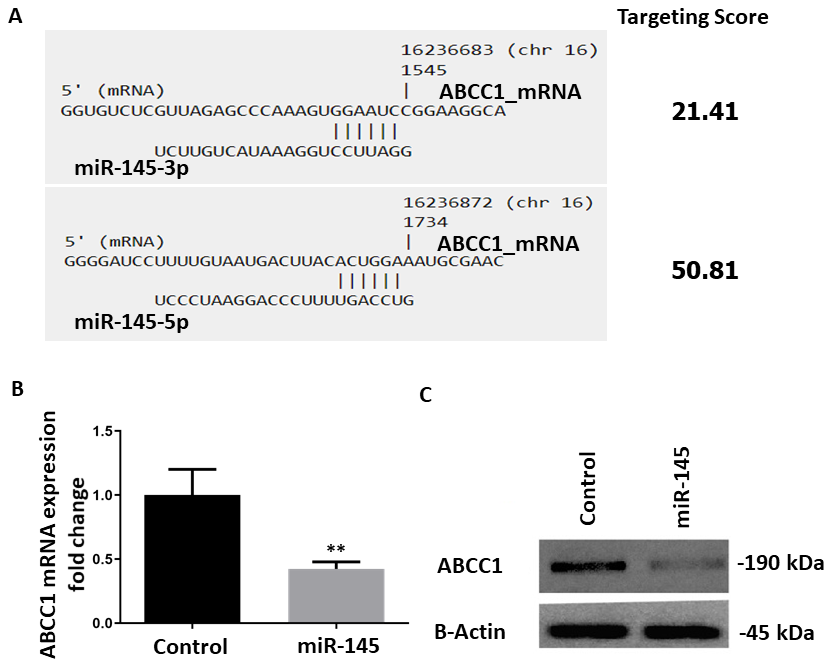


**Fig. 5. MiR-145 targets ABCC1 mRNA.** Target prediction analysis showed both miR-145-3p and -5p recognize binding sites within ABCC1 mRNA, with prediction scores of 21.41 and 50.81, respectively (A). ABCC1 mRNA expression was significantly reduced in cells transfected with miR-145 compared with the blank control (B). Consistently, ABCC1 protein levels were decreased in the group receiving combined oxaliplatin and miR-145 treatment compared with the blank control, with β-actin used as a loading control (C). ****P < 0.0001.


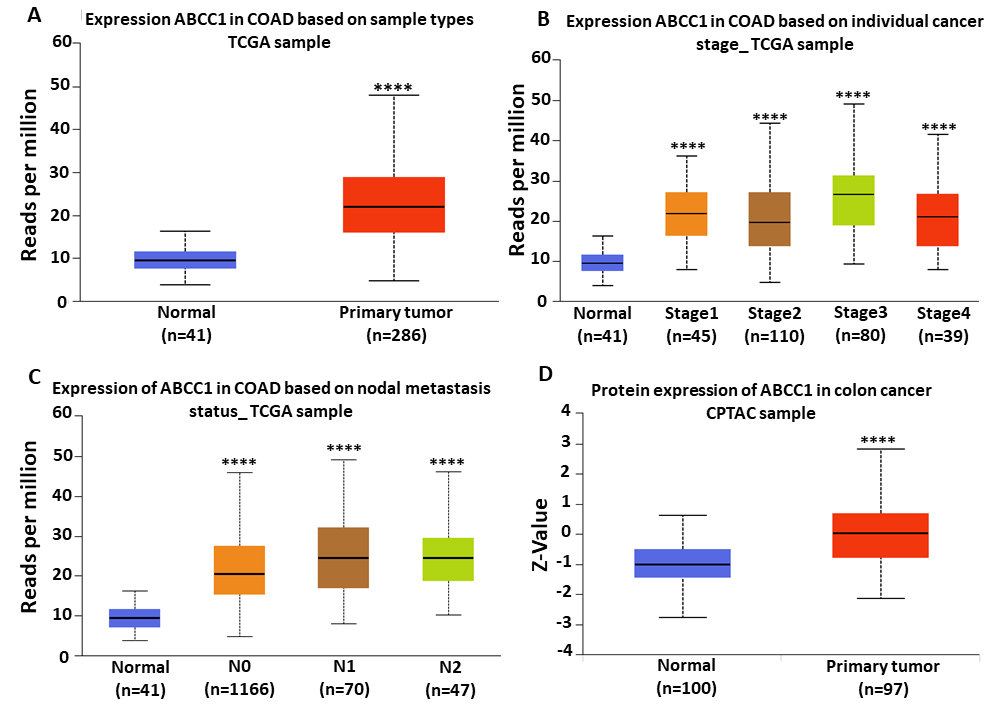


**Fig.6. ABCC1 overexpressed in colon adenocarcinoma.** Data from the TCGA-COAD project shows that ABCC1 expression is significantly increased in primary tumors tissues compared with adjacent normal tissues (A). ABCC1 expression level significantly increased across tumor stages I–IV (B), and N0-N2 tissues compared to adjusted normal tissues(C). ABCC1 Protein expression was increased in colon cancer tissues compared to adjusted normal tissues from TCGA and the Cancer Institute’s Clinical Proteomic Tumor Consortium (CPTAC) samples (D). ****P < 0.0001.
