# Supplementary figures and images for: miR-145 restoration overcomes oxaliplatin resistance via ABCC1 in colorectal cancer
Source: J Egypt Natl Canc Inst. 2026 May 25;38:17. doi: 10.1186/s43046-026-00349-8 (PMC13313293; doi:10.1186/s43046-026-00349-8)

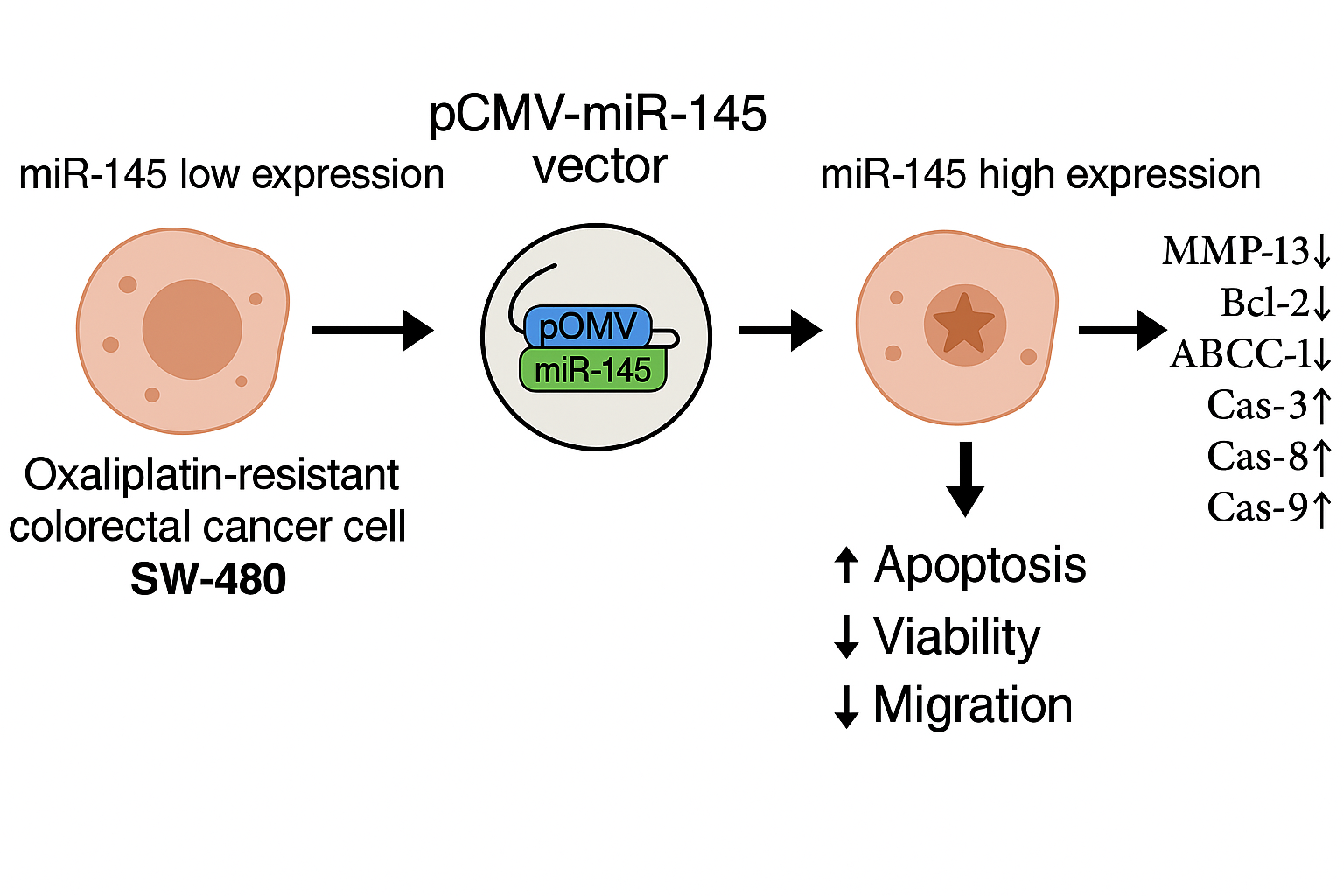

Supplement: Supplementary file 2 — Supplementary Material 2. [file 43046_2026_349_MOESM2_ESM.tiff]

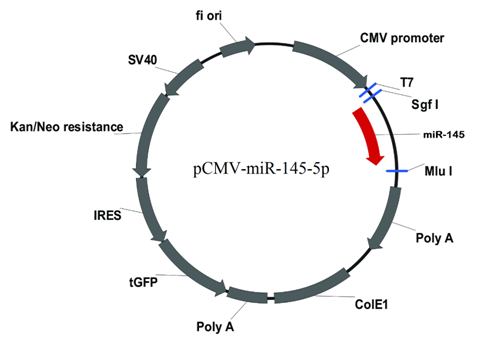

Supplement: Supplementary file 3 — Supplementary Material 3. [file 43046_2026_349_MOESM3_ESM.zip › Figure 1.tiff]

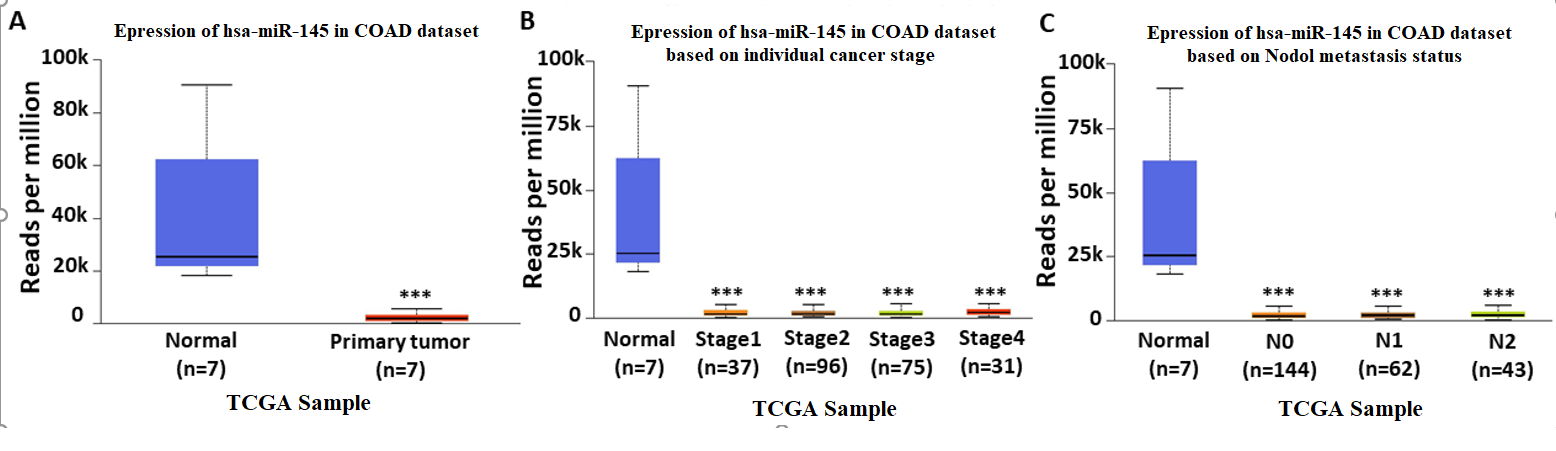

Supplement: Supplementary file 3 — Supplementary Material 3. [file 43046_2026_349_MOESM3_ESM.zip › figure 2.tiff]

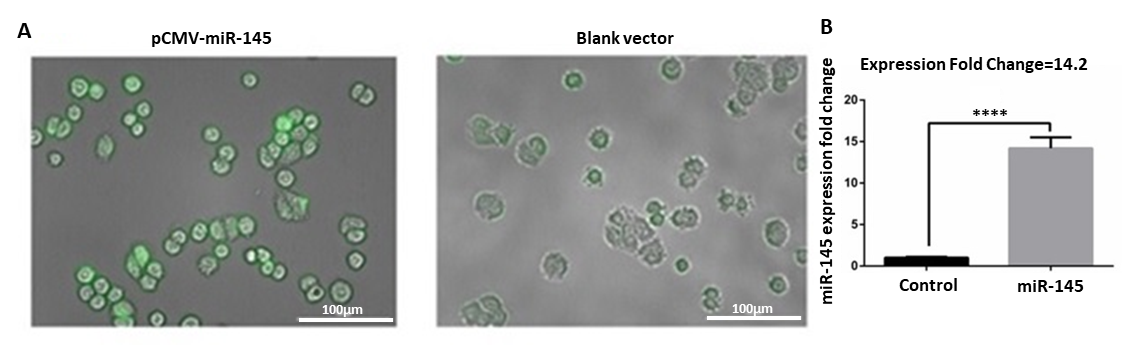

Supplement: Supplementary file 3 — Supplementary Material 3. [file 43046_2026_349_MOESM3_ESM.zip › Figure 3.tiff]

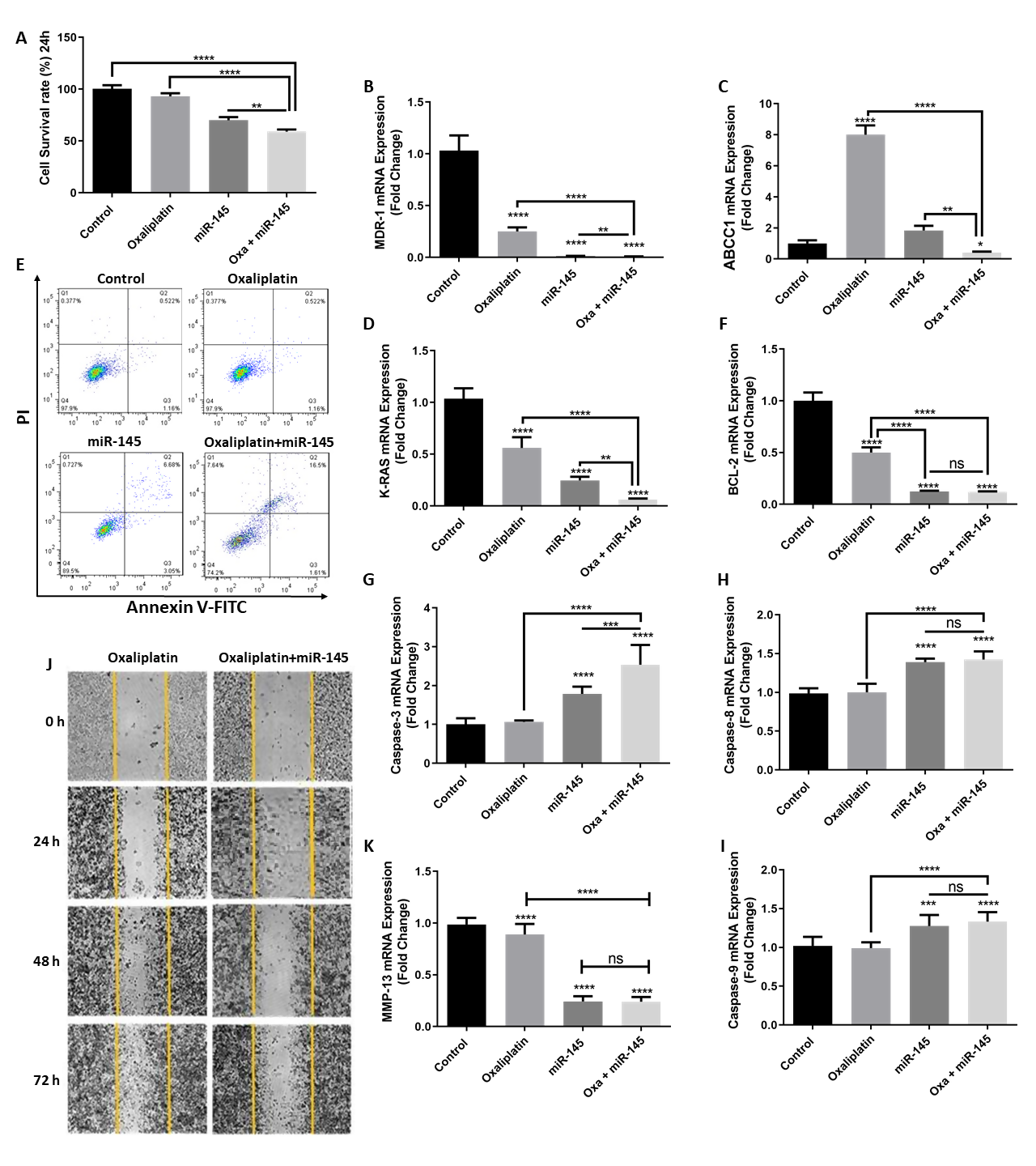

Supplement: Supplementary file 3 — Supplementary Material 3. [file 43046_2026_349_MOESM3_ESM.zip › Figure 4.tiff]

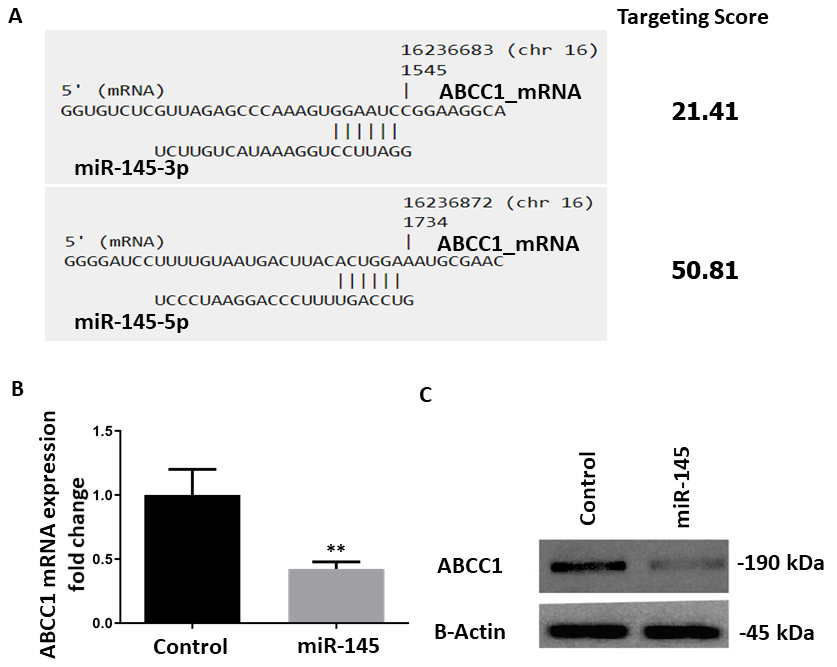

Supplement: Supplementary file 3 — Supplementary Material 3. [file 43046_2026_349_MOESM3_ESM.zip › Figure 5.tiff]

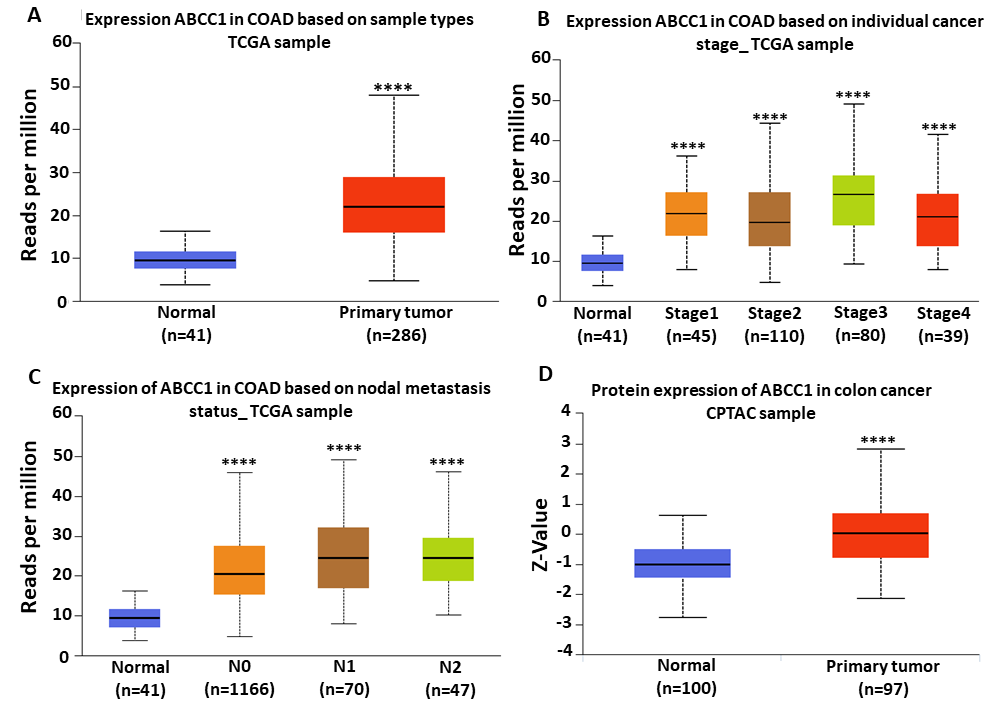

Supplement: Supplementary file 3 — Supplementary Material 3. [file 43046_2026_349_MOESM3_ESM.zip › Figure 6.tiff]
